# Eye Movement Patterns Can Distinguish Schizophrenia From the Major Affective Disorders and Healthy Control Subjects

**DOI:** 10.1093/schizbullopen/sgac032

**Published:** 2022-05-20

**Authors:** David St Clair, Graeme MacLennan, Sara A Beedie, Eva Nouzová, Helen Lemmon, Dan Rujescu, Philip J Benson, Andrew McIntosh, Mintu Nath

**Affiliations:** Division of Applied Medicine, Institute of Medical Sciences, University of Aberdeen, Aberdeen, UK; Clinical Research Centre, Royal Cornhill Hospital, Aberdeen, UK; Centre for Healthcare Randomised Trials (CHaRT), University of Aberdeen, Aberdeen, UK; Clinical Research Centre, Royal Cornhill Hospital, Aberdeen, UK; Clinical Research Centre, Royal Cornhill Hospital, Aberdeen, UK; Clinical Research Centre, Royal Cornhill Hospital, Aberdeen, UK; Department of Psychiatry, Medical University of Vienna, Vienna, Austria; Department of Psychology, University of Aberdeen, Aberdeen, UK; Division of Psychiatry, Royal Edinburgh Hospital, Edinburgh, UK; Medical Statistics Team, Institute of Applied Health Sciences, University of Aberdeen, Aberdeen, UK

**Keywords:** eye movements, schizophrenia, affective disorders, biomarker, predictive modelling

## Abstract

**Background and hypothesis:**

No objective tests are currently available to help diagnosis of major psychiatric disorders. This study evaluates the potential of eye movement behavior patterns to predict schizophrenia subjects compared to those with major affective disorders and control groups.

**Study design:**

Eye movements were recorded from a training set of UK subjects with schizophrenia (SCZ; *n* = 120), bipolar affective disorder (BPAD; *n* = 141), major depressive disorder (MDD; *n* = 136), and healthy controls (CON; *n* = 142), and from a hold-out set of 133 individuals with proportional group sizes. A German cohort of SCZ (*n* = 60) and a Scottish cohort of CON subjects (*n* = 184) acted as a second semi-independent test set. All patients met DSMIV and ICD10 criteria for SCZ, BPAD, and MDD. Data from 98 eye movement features were extracted. We employed a gradient boosted (GB) decision tree multiclass classifier to develop a predictive model. We calculated the area under the curve (AUC) as the primary performance metric.

**Study results:**

Estimates of AUC in one-versus-all comparisons were: SCZ (0.85), BPAD (0.78), MDD (0.76), and CON (0.85). Estimates on part-external validation were SCZ (0.89) and CON (0.65). In all cases, there was good specificity but only moderate sensitivity. The best individual discriminators included free viewing, fixation duration, and smooth pursuit tasks. The findings appear robust to potential confounders such as age, sex, medication, or mental state at the time of testing.

**Conclusions:**

Eye movement patterns can discriminate schizophrenia from major mood disorders and control subjects with around 80% predictive accuracy.

## Introduction

The current categorical diagnostic classification system in psychiatry relies upon face validity. There are no objective tests to support clinical diagnosis, monitor the progress of illness, or inform choice of treatment.^1,2^ Possible reasons are discussed, but no consensus has emerged.^3–5^ At the core of the current classification system lies the hundred-year old so-called Kraepelinian dichotomy that sets a boundary between schizophrenia and major mood disorders. Unfortunately, it remains controversial whether these disorders are qualitatively different from each other.^6,7^ Evidence from clinical and molecular genetics shows considerable genetic overlap suggesting a continuum,^8–12^ whereas a recent long-term clinical outcome study comparing nonaffective with affective psychosis does not support the idea of a continuum.^13^

Biomarker discovery efforts in psychiatry have mostly focused on neuro-imaging and genomics. While some interesting results have emerged, most studies use binary case-control methodologies, replications are few and robust modelling approaches, and appropriate validations are rare.^14–19^.

Atypical eye movements in unmedicated psychiatric patients were first reported in 1908.^20^ Since then, a considerable literature has consistently reported eye movement abnormalities in schizophrenia patients compared to healthy controls, reviewed by Wolf et al.^21^ The literature for other major psychiatric disorders such as unipolar and bipolar disorders is much more modest with often inconsistent findings, small sample sizes, and few studies have compared eye movements in mood disorders with schizophrenia. The most common findings are abnormalities of pursuit in all diagnostic groups with schizophrenia patients most impaired.^22–26^

Most recently, Clementz et al replicated earlier studies using a test battery that included neuropsychology, eye movements, EEG in a large cohort, and demonstrated excellent discrimination between psychosis and control populations, but none of the physiological biomarkers differentiated the psychosis subgroups.^27^ In general, however, results have not shown sufficient sensitivity or specificity to be of clinical value.

We earlier reported that individuals with schizophrenia show significant eye movement differences from a mentally healthy comparison group when performing image viewing, smooth pursuit, and steady fixation tasks. Indeed using a gradient boosted (GB) decision tree machine learning algorithm, we could distinguish new schizophrenia cases from controls with around 80% predictive accuracy.^28^ These results have now been independently replicated.^29^ Using similar protocols, the authors derived an integrated score from the same eye movement tasks and found they could separate schizophrenia from controls with 82% accuracy.

In this study, we have expanded our schizophrenia and control cohorts and tested two new large groups of individuals with bipolar and recurrent unipolar affective disorder. We hypothesized that if eye movement viewing patterns could discriminate schizophrenia from major mood disorders as well as from mentally unaffected control subjects, they would have potential clinical utility as biomarkers to assist with the diagnosis of schizophrenia.

We have also examined in detail the effects of potential confounders including age and effects of psychotropic medication. Recent large studies by Coors et al^30^ and Takahashi et al^31^ highlight the importance of the former as a potential confounder in eye movement studies.

## Methods

### Participants

Data from 672 subjects constituted the main dataset for calibrating and validating the classifier (see later). The data included healthy control (CON; *n* = 177) individuals along with patients diagnosed with schizophrenia (SCZ, *n* = 150) including 64 schizophrenia patients described in the earlier study,^28^ bipolar affective disorder (BPAD; *n* = 176), and unipolar major depressive disorder (MDD; *n* = 169). We additionally used a German cohort of SCZ (*n* = 60),^28^ and a Scottish cohort of CON subjects (*n* = 184) as a semi-independent second validation dataset (see later). All Scottish patients were identified through the psychiatric services of local NHS Trusts. Diagnoses were initially made on ICD-10 criteria by attending clinicians and then independently confirmed by the research team through examination of psychiatric case notes, OPCRIT checklist, and a structured clinical interview for DSM-IV.^32–34^

All met DSM-IV and ICD-10 criteria for schizophrenia, bipolar disorder, or unipolar depression. The latter was defined as having at least two major depressive episodes or chronic depression lasting over 18 months. All depression cases were ascertained through psychiatric care services. Mentally healthy nonclinical controls were recruited mainly through public advertisements and also included a volunteer panel at the University of Aberdeen.

The Scottish and German studies obtained full multi-regional ethics committee (MREC) and institutional review board (IRB) approvals, respectively, and were conducted in accordance with the Declaration of Helsinki.

### Phenotype Measures

Following informed consent, a brief interview and collection of demographics, eye movements were recorded on all subjects. Static image free viewing, smooth pursuit, and steady fixation tasks were administered. The protocol has been previously described.^22,28^ Minor changes were introduced in the recording protocols during the 12-year period of the study. However, we found minimal evidence of incompatibility arising from these changes and proceeded to analyze the data as a single dataset. The eye movement data were quality controlled and then scored semi-automatically offline as previously described.^2,28^ This generated a total of 98 eye movement variables (aka features); a complete list of these variables is presented in the supplement (Supplementary Table 2). These eye movement variables along with sex were considered for the development of the multiclass classifier (discussed below).

Following eye movement recording, a diagnostic interview using SCID or MINI was administered to all cases and controls together with a brief illness-agnostic neuropsychological test battery ^22^. Neuropsychology results will be reported elsewhere.

### Statistical Analysis

We partitioned the main dataset (*n* = 672) into training (Train, *n* = 539; 80% of data) and testing or hold-out (Test-1, *n* = 133; 20% of data) datasets by randomly selecting individuals from all four groups ensuring that representation in both datasets was similar to the whole cohort. We used the test dataset (Test-1) for validation-1 and the semi-independent dataset (60 SCZ and 184 CON, Test-2) for validation-2. Missing values for any features in the training dataset were imputed using the bootstrap aggregation-based approach.

**Fig. 1.**
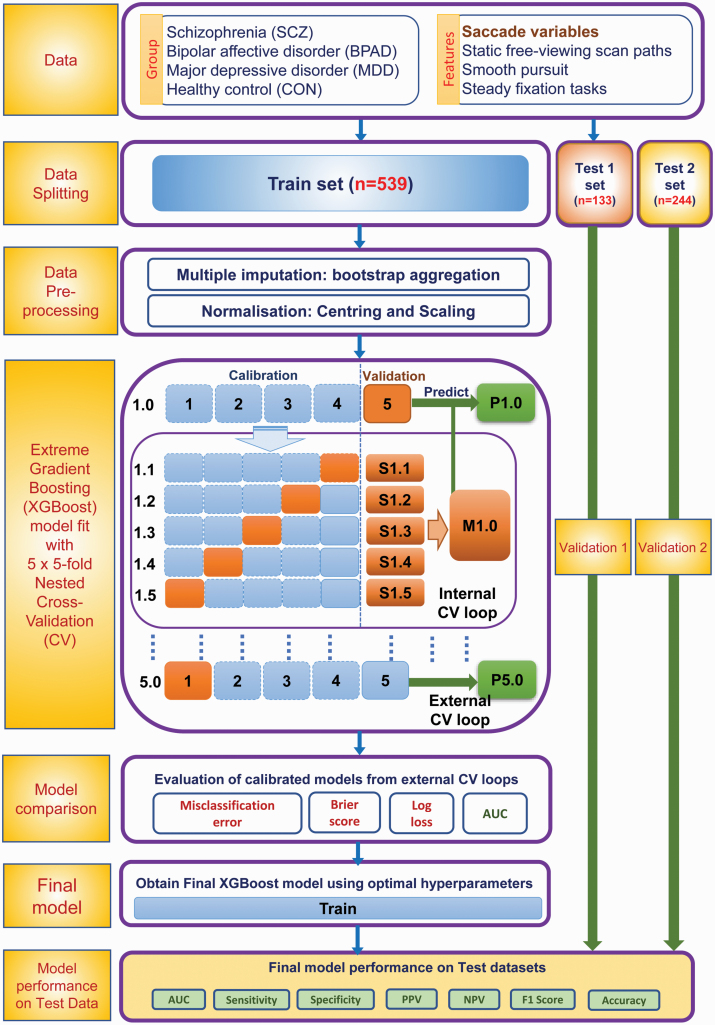
Workflow pipeline for multiclass classifier to predict psychiatric disorders using eye movement data.

We employed a gradient boosted (GB) decision tree multiclass classifier to develop a predictive model of major psychiatric disorders using eye movement features in a machine-learning (ML) framework. To develop a robust, reproducible, and accurate classifier, we implemented a modified version of the 5 × 5 fold nested cross-validation (CV) scheme outlined before.^35,36,37^ Figure 1 gives a schematic representation of the full pipeline. A detailed description of machine learning outflow is provided in the Supplementary Material.

The performance of the final classifier was evaluated on both validation datasets (Test-1, Test-2) using a range of performance metrics. We calculated the area under the curve (AUC) as the primary performance metric using three methods: the generalized overall AUC, one-versus-all (OVA) or one-versus-rest for each group, and pairwise AUCs using one-versus-one (OVO).^36^ Other performance metrics included: sensitivity, specificity, positive predictive value (PPV), negative predictive value (NPV), F1 score, accuracy and balanced accuracy under OVA using the 50% cutoff probability. We conducted the bootstrap sampling with 1000 replicates to calculate the 95% confidence interval of these performance metrics.

### Potential Confounders

We considered demographic, behavioral, and other clinical factors that could potentially influence eye tracking performance. These included: age, caffeine consumption, nicotine use, anxiety, and depression subscores at time of testing (based on the Hamilton Anxiety and Depression Scale or HADS questionnaire) and current psychotropic medication. To assess the confounding effect of age, we developed two classifiers with eye movement data—including and excluding the age variable—and compared their performance metrics.

To further evaluate the importance of potential confounding variables, we conducted a principal component (PC) analysis of 98 eye movement features of all 672 participants and computed PC scores of each participant for the first 20 PCs, which captured approximately 80% variability of the eye movement data. We then fitted a separate single variable PC regression model by regressing the PC scores on each potential confounding variable and estimated the coefficient of determination (R^2^) representing the proportion of variability explained by the confounding variable.

## Results

### Clinical Samples

The demographic characteristics of the groups used for training the classifier are shown in Table 1. Demographics for the two test datasets are presented in Supplementary Table 1. The bipolar group was similar to the schizophrenia group, but the average age was older. The patients in the recurrent unipolar depression group were older than the bipolar group. The amount and type of medication varied both within and between groups; almost all schizophrenia patients were receiving neuroleptic medication while bipolar patients received mood stabilizers and/or antidepressants, with 30% also prescribed neuroleptics. All save five recurrent unipolar subjects were prescribed psychotropic medication; these included antidepressants and in a minority of cases also mood stabilizers and/or anxiolytics.

**Table 1.** Demographics of the Patient and Control Subjects Used for Developing the Classifier. Demographics of Others Used for Validating the Classifier (Test-1 and Test-2) Can be Found in Supplementary Table 1

	Schizophrenia (SCZ)	Bipolar Affective Disorder (BPAD)	Major Depression Disorder (MDD)	Healthy Control (CON)
**Training data (Train; *N* = 539)**				
*N*	120	141	136	142
Sex,	35:85	81:60	86:50:00	84:58:00
Female:Male				
Age (years),	43.0	47.0	46.5	28.0
Median (Q1, Q3)	(32.8, 51.2)	(38.0, 55.0)	(36.0, 57.0)	(23.0, 45.0)
Education (years),	12.0	15.0	13.5	15.0
Median (Q1, Q3)	(9.5, 15.0)	(12.0, 15.0)	(10.0, 15.0)	(13.5, 16.0)
Illness age of onset (years),	22.0	27.0	30.0	
Median (Q1, Q3)	(19.0, 28.0)	(21.0, 35.0)	(23.0, 41.0)	
Illness duration (years),	19.0	17.0	11.0	
Median (Q1, Q3)	(13.8, 27.5)	(9.0, 26.0)	(6.0, 21.0)	
CPZ,	525.0	50.0	0.0	
Median (Q1, Q3)	(237.5, 862.5)	(0.0, 150.0)	(0.0, 33.2)	
Nicotine (cigarettes/day),	5.0	0.0	0.0	0.0
Median (Q1, Q3)	(0.0, 20.0)	(0.0, 10.0)	(0.0, 0.0)	(0.0, 0.0)
Caffeine intake (cups/day),	4.0	4.0	4.0	3.0
Median (Q1, Q3)	(2.2, 7.0)	(2.0, 5.0)	(3.0, 6.0)	(1.0, 5.0)
HADS Anxiety,	8.0	8.0	11.0	4.0
Median (Q1, Q3)	(6.0, 12.0)	(4.0, 12.0)	(7.0, 14.0)	(2.0, 6.0)
HADS Depression,	7.0	5.0	8.0	1.0
Median (Q1, Q3)	(4.0, 9.0)	(2.0, 10.0)	(5.0, 12.0)	(0.0, 3.0)

*Note:* CPZ, Chlorpromazine equivalents (mg/day). Q1, Q3 are first and third quartiles. Education indicates years in full time education.

### Eye Movement Variables

We considered a total of 98 eye movement variables broadly consisting of different summarized measurements within the domains of free-viewing, fixation stability, and smooth pursuit activities. Supplementary Table 2 presents the description of all the eye movement variables and Supplementary Table 3 provides summary statistics (median, Q1, Q3, minimum, and maximum) based on the full (Train + Test-1) population. Supplementary Figure 1 illustrates an overview of the estimates of correlation coefficients between the variables. In general, variables within the same domain showed positive correlation while variables of different domains showed poor correlation. Variables related to fixation frequency and fixation duration within the smooth pursuit domain showed a strong negative correlation.

### Performance Measures of Gradient Boosting Multiclass Classifier

**Fig. 2.**
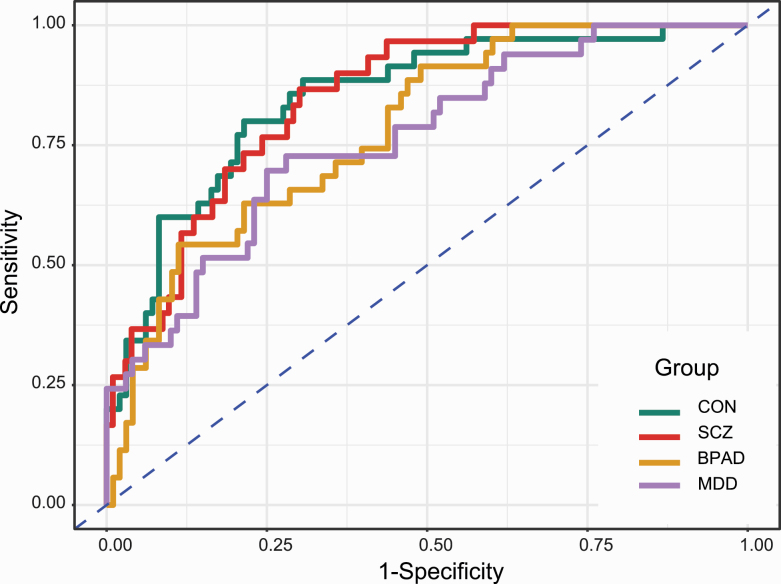
Receiver operating characteristic (ROC) curves of schizophrenia (SCZ), bipolar affective disorder (BPAD), major depressive disorder (MDD), and healthy control (CON) based on validation of the fitted multiclass classifier on Test-1 dataset under One-versus-All (OVA) scenario.

Different performance metrics of the classifier on Test-1 and Test-2 datasets along with the corresponding confusion tables are presented in Tables 2 and 3. The receiver operating characteristic (ROC) plots of four groups under OVA are presented in figure 2. For Test-1, the overall generalized estimate of AUC was 0.81. Estimates under OVA ranged from 0.76 to 0.85 while estimates under OVO ranged from 0.82 from 0.82 to 0.85 except for BPAD vs MDD, which was 0.69. For Test-2, estimates of OVA for CON and SCZ were 0.64 and 0.89, respectively, while the estimate of OVO between CON and SCZ was 0.77. On the Test-1 dataset, the fitted gradient boosted multiclass classifier correctly predicted 57%, 60%, 49%, and 55% patients of CON, SCZ, BPAD, and MDD while for the Test-2 dataset, the classifier correctly predicted 63% and 58% of patients of CON and SCZ group using the notional cutoff probability of 50%. The representation of four groups ranged from 23% to 26% in Test-1 dataset. The specificity of the classifier was over 82% in the Test-1 dataset, while it was over 63% in the Test-2 dataset. The overall accuracy of the classifier was 0.55 (95% confidence interval, 0.46, 0.64) for the Test-1 and 0.61 (95% confidence interval, 0.55, 0.68) for the Test-2. Supplementary Table 4 provides detailed performance metrics and the 95% confidence interval (lower, upper) of the classifier on both validation datasets obtained from bootstrap sampling using 1000 replicates.

**Table 2.** Confusion Matrix and Estimates of Area Under the Curve (AUC) Based on Validations of the Fitted Multiclass Classifier on Two Test Datasets

Validation on Test-1	Reference						AUC^b^		
Prediction	CON	SCZ	BPAD	MDD	Group	CON	SCZ	BPAD	MDD
CON	**20**	4	4	6	CON	**0.85**			
SCZ	7	**18**	5	3	SCZ	0.84	**0.85**		
BPAD	4	3	**17**	6	BPAD	0.85	0.82	**0.78**	
MDD	4	5	9	**18**	MDD	0.82	0.84	0.69	**0.76**
**Validation on Test-2** ^a^	**Reference**						**AUC** ^b^		
**Prediction**	CON	SCZ	BPAD	MDD	**Group**	CON	SCZ		
CON	**115**	17			CON	**0.64**			
SCZ	24	**35**			SCZ	0.77	**0.89**		
BPAD	31	1							
MDD	21	0							

*Note:* CON, Healthy Control; SCZ, Schizophrenia; BPAD, Bipolar Affective Disorder; MDD, Major Depressive Disorder.

^a^The validation on Test-2 dataset does not have any representation of BPAD and MDD patients.

^b^AUC table represents the overall groupwise AUC by one-versus-all (OVA) (diagonal element) and pairwise AUCs by one-versus-one (OVO) methods (off-diagonal elements).

The bolds in the table on the left confusion matrix are the correctly classified cases,on the AUC right side the bold are one versus all comparisons,the non bold are One versus one comparisons.

**Table 3.** Performance Metrics Based on Validations of the Fitted Multiclass Classifier on Two Test Datasets

Statistics	CON	SCZ	BPAD	MDD
**Validation on Test-1**				
Sensitivity	0.57	0.60	0.49	0.55
Specificity	0.86	0.85	0.87	0.82
Positive Predictive Value (PPV)	0.59	0.55	0.57	0.50
Negative Predictive Value (NPV)	0.85	0.88	0.83	0.85
F1 Score	0.58	0.57	0.52	0.52
Accuracy	0.78	0.80	0.77	0.73
Balanced Accuracy	0.71	0.73	0.68	0.68
**Validation on Test-2** ^a^				
Sensitivity	0.63	0.58		
Specificity	0.60	0.91		
Positive Predictive Value (PPV)	0.83	0.67		
Neg Predictive Value (NPV)	0.34	0.87		
F1 Score	0.71	0.63		
Accuracy	0.62	0.83		
Balanced Accuracy	0.61	0.75		

*Note:* CON, Healthy Control; SCZ, Schizophrenia; BPAD, Bipolar Affective Disorder; MDD, Major Depressive Disorder. Further details on performance metrics are provided in Supplementary Table 4.

^a^The validation on Test-2 dataset does not have any representation of BPAD and MDD patients.

### Important Eye Movement Variables

The best individual discriminators included free viewing, fixation duration, and smooth pursuit tasks. Complete information of variable importance for all 98 eye movement variables and sex is provided in Supplementary Table 5.

### Effects of Potential Confounders

The classifier including age showed very modest changes in the performance metrics when compared with the classifier excluding age. For example, the estimates of AUC for SCZ vs other groups increased from 0.85 to 0.86 when age was included as a feature in the classifier (Supplementary Table 6). The complete performance measurements of the classifier including age and eye movement features are presented in Supplementary Tables 6-8. Supplementary Table 10 shows the age variable included in the principal component regression analyses. Results show a marginal influence of age on saccade variables (9.6% on PC1, 6.1% on PC4, and 1.7% on PC7). Age explains approximately 2.9% of the variability in saccade features. The variable importance for sex was low (ranked 84th for the classifier excluding age, and 67th for the classifier including age).

Psychotropic medication effects were the most important potential confounders. Since almost all individuals with schizophrenia were receiving neuroleptics, and among the rest, only a minority with BPAD were prescribed neuroleptics, a direct comparison across groups was not possible. By contrast, almost all affective disorder subjects were prescribed antidepressant medications. Based on principal component regression models of the first 20 PC scores that represent approximately 80% variability of eye movement data, we observed that psychotropic medications contributed 3.3% of the variability in the data. In contrast, other confounders like caffeine consumption and nicotine use, anxiety, and depression subscores from the HADS questionnaire, explained a negligible amount of variability (range from 0.3% to 0.7%) in the eye movement data. Supplementary Table 9 presents additional summary statistics of different confounders. We explored the clinical and demographic features of the 12 test schizophrenia cases wrongly classified and the 15 nonschizophrenia subjects missclassified as schizophrenia on the four-way comparisons. No consistent pattern emerged among the 12 missclassified schizophrenia cases, but 3 of 5 bipolar cases misclassified as schizophrenia had severe or very severe illness eg, on depot medication or spent many years in hospital. Four of 7 control cases had minor anomalies, astigmatism, restlessness, and calibration difficulties during testing, and very odd personality. Supplementary Table 11 gives the predictive probability scores of all 133 test subjects using four-way comparisons. Of 133 test cases, the average predictive probability of correctly classified cases was 0.6976 and missclassified cases 0.5882; for schizophrenia cases, average predictive probability was 0.6847 for correctly diagnosed and 0.5530 for misclassified cases.

## Discussion

Our results demonstrate that eye movement behavior patterns discriminate schizophrenia from unipolar and bipolar affective disorders and unaffected controls. We validated the classifier using the hold-out (Test-1) and semi-independent (Test-2) datasets for validations. Test-1 showed an average Area Under Curve (AUC) of 0.84 when the schizophrenia group was compared to the three other groups combined. Test-2 also supported our findings with an overall AUC of 0.77. An AUC of around 0.80 is generally considered good predictive performance.^38,39^ Pairwise comparisons of schizophrenia confirmed similar predictive ability. Bipolar and MDD patients also performed well for the one-versus-all (OVA) comparisons but indicated comparatively lower discrimination on direct comparisons with each other (see Tables 2 and 3 and figure 2). Based on the 50% cutoff probability, the confusion matrices presented similar levels of discrimination and accuracy on both validation sets. The overall accuracy of 61%, as observed, is considerably better than the estimates of prevalence (23% to 26%) of four groups in the Test-1 dataset or that expected by chance (ie, 25%).

Except for AUC, performance metrics like sensitivity, specificity, positive and negative predictive value etc. are the outcomes from the OVA comparison with the notional cut-off probability set at 0.50. Naturally, estimates of performance metrics are conditional on the choice of the cut-off value; this choice generally incorporates the information from the ROC data with the utility-based decision theory to identify the optimal cut-off point in a practical scenario. For example, the decision may include the disease prevalence, maximising sensitivity or specificity, maximising accuracy, cost of false positive, or negative results etc., accounting for how the model is to be used for the decision-making. On the other hand, ROC curve analysis and AUC-based estimates presented here have several advantages. AUC is an effective and combined measure of sensitivity and specificity that describes the inherent validity of the diagnostic test to discriminate between positive and negative populations. It is not influenced by decision criteria and the prevalence of the disease. AUC estimate is an ideal quantitative measurement that supports comparing different tests and combining multiple tests to improve diagnostic accuracy.^38,39^ Therefore, AUC is a reliable performance metric for the diagnostic test, and AUC estimates obtained from the model demonstrate good discriminating ability between three psychological disorders and unaffected controls.

Although the overall performance metrics are encouraging, we acknowledge a model developed on larger sample sizes integrated with rigorous decision criteria are required to upgrade the model from the current levels of modest sensitivity and good specificity to the point where the model would demonstrate clinical utility. However, to our knowledge, this is the first large study to document the good model-based predictive performance of eye movement patterns of healthy controls vs schizophrenia and two other major mood disorders.

Elements of the free viewing and fixation tasks best distinguished individuals with schizophrenia from other groups; they produced restricted viewing patterns when looking at static images and had difficulty inhibiting saccades towards a distractor during the steady fixation task. Individuals with mood problems exhibited faster saccades than other groups during free viewing, and bipolar cases also produced restricted fixation activity distinct from controls but not as pronounced as in the schizophrenia group. Subtle differences in smooth pursuit performance differentiated affective disorders from other groups. We did not try to weigh the discriminatory importance of the variables for each disorder separately in the four-way comparisons.

We observed that psychotropic medication contributed a modest 3.3% of the variability in eye movement behavior patterns. This finding suggests that medication through reverse causation is unlikely to be responsible for differences between patient groups. An extensive literature on eye movement abnormalities in medication-free schizophrenics and high-risk relatives supports this view.^40^ We have previously reported eye movement patterns in a small number of schizophrenia patients medication-free at the time of testing were similar to medicated schizophrenia patients.^28^ No new medication-free schizophrenia patients were available in the current study. We did, however, observe a minimal association between the amount of chlorpromazine equivalents and the primary eye movement patterns using principal component analysis (see Supplementary Table 10). Similar conditions applied to affective disorders. Almost all patients received antidepressants and or mood-stabilizing medications. However, the main abnormalities compared to controls were in measures of smooth pursuit, and the literature suggests relative independence of abnormal smooth pursuit eye movements from antidepressant and neuroleptic medications.^40,41^

The affective disorders groups in this study were for comparison purposes only. The majority (ca. 65%) of bipolar cases were bipolar I, namely those that share many clinical features with schizophrenia. All MDD cases were under psychiatric supervision and represented the severer end of the MDD spectrum. Mental state at the time of testing MDDs varied across subjects, with most in partial remission of symptoms. The sample sizes of unipolar and bipolar cases are sufficient to be confident that schizophrenia cases can be distinguished from them with a high degree of accuracy. By contrast, the bipolar and unipolar cohorts showed good separability from control subjects but not from each other. Larger sample sizes are required to determine if eye movement behavior patterns can stratify affective disorders as a whole into clinically useful subgroups such as bipolar patients with and without psychosis. It remains to be seen whether cases with noncore or missing features of the disorders will reveal the same differences, thus delineating the approximate core diagnostic category.^42^ It is essential to know the time of first atypical eye movement patterns and its importance for the early diagnosis of individuals at high risk of major mental health problems.

### Strengths and Limitations

Our multiclass classifier model of eye movement behavior patterns distinguished schizophrenia from bipolar and unipolar disorder and controls with good predictive performance. All cases met DSMIV operationalized criteria. It is especially encouraging that all MDD cases were ascertained through psychiatric services. These patients with moderate to severe depression are clinically more difficult to differentiate from other forms of major mental illness. The sample sizes for all groups were reasonably large, balanced, and internally consistent. The relatively advanced age of the unipolar cohort also makes it probable that few will convert at a later date to a bipolar diagnosis. In contrast to many current predictive neuro-imaging and genetic studies in psychiatry, we validated our classifier using two validation datasets. As in our earlier article for predictive performance, we used a GB modelling framework on these much-expanded schizophrenia and control cohorts. The findings for schizophrenia vs unaffected controls groups are essentially unchanged from our earlier findings and those of Morita et al.^28,29^

We implemented a rigorous machine learning pipeline to develop a robust, reproducible, and accurate gradient boosted multiclass classifier and validated the classifier using two validation datasets. The nested cross-validation framework separated the classifier learning task from the calibration task and accurately estimated performance metrics by averaging across folds, hence the setup allowed limiting the overfitting and yielded robust tuning of the postprocessing algorithm.^35,36^ We adopted a transparent approach to model the data and provided a detailed pipeline to account for samples, features, missing value imputation, hyperparameter search, and algorithms, therefore, enhancing the reproducibility of results.^35^ We obtained the 95% confidence intervals of all performance metrics using a rigorous bootstrap-based approach.

The study also has some limitations. To our knowledge, we used the largest sample of this kind available and adopted rigorous strategies to limit the overfitting: however, further calibration of the model with larger training samples is necessary to try to improve sensitivity. We could not conduct full external validation of the model due to the nonavailability of all phenotypes. Test-1 and Test-2 controls were recruited from the same Scottish study and followed the same experimental protocol. We also used data from the German schizophrenia subjects exclusively for external validation. Although we previously reported the German data in a different modelling context we considered them a semiindependent group because they were recruited through Ludwig Maximilian University, but were tested using the same protocols by the Aberdeen research staff on temporary secondment to Munich. All eye movement data for the affective disorders comparative groups were not reported earlier. The psychiatric conditions we studied are all highly heterogeneous and overlap clinically: a discrimination much higher than AUC of 0.8 would raise concerns of overfitting of the training data, and much smaller value would have minimal clinical value. There are also, as Moriarity et al^43^ indicate, many unobserved issues of measurements of noninvariance in biological psychiatry that can complicate otherwise promising findings. In these circumstances, Onitsuka et al^44^ highlight there is an urgent need for replication of promising eye movement findings in psychiatry in large multicentred studies.

## Conclusions

Eye movement behavior patterns can distinguish schizophrenia from major affective disorders and unaffected controls with a reasonable degree of accuracy. Although the performance characteristics based on AUC are in the 80% range, most of this is accounted for by good specificity, and only modest sensitivity. Larger sample sizes integrated with robust decision criteria will be needed to obtain a clear picture of the biological significance of our findings and whether they may prove useful in a clinical setting.

## Supplementary Material

sgac032_suppl_Supplementary_Material

## Data Availability

Requests for access to anonymized study data for replication or related studies should be directed to the corresponding author. All reasonable requests will be considered positively. There are plans for the datasets to be publically posted in due course.
