# Supplementary material for: Eye Movement Patterns Can Distinguish Schizophrenia From the Major Affective Disorders and Healthy Control Subjects
Source: Schizophr Bull Open. 2022 May 20;3(1):sgac032. doi: 10.1093/schizbullopen/sgac032 (PMC9155263; doi:10.1093/schizbullopen/sgac032)
Supplement: sgac032_suppl_Supplementary_Material [file sgac032_suppl_Supplementary_Material.pdf]

Eye movement patterns can distinguish Schizophrenia from the major affective disorders and healthy control subjects

Supplementary Materials

Contents

Machine learning workflow 2

Table 1: Demographics of the patient and control subjects used for validating the classifier (Test-1 and Test-2). 4

Supplementary Table 2. Details of eye movement variables 6

Supplementary Table 3. Summary statistics of eye movement variables used in the study (N=672)... 11

Supplementary Figure 1. Correlation plot of all eye movement variables (N=672) 16

Supplementary Table 4. Detailed performance metrics along with the 95% confidence interval (lower, upper)† based on validations of the fitted gradient boosted multiclass classifier (including all eye movement features and sex) on Test-1 (N=133) and Test-2ǂ (N=244) datasets 17

Supplementary Table 5. Variable importance of the fitted gradient boosted multiclass classifier (including all eye movement features and sex) and corresponding median (Q1, Q3) of all eye movement variables in Test 1 data (N=133). 19

Supplementary Table 6. Confusion matrix and estimates of area under the curve (AUC) based on validations of the fitted gradient boosted multiclass classifier (including age, sex and all eye movement features) on Test-1 (N=133) and Test-2ǂ (N=244) datasets 23

Supplementary Table 7. Performance metrics based on the validation of the fitted gradient boosted multiclass classifier (including age, sex and all eye movement features) on Test-1 (N=133) and Test-2ǂ (N=244) datasets. 24

Supplementary Table 8. Detailed performance metrics along with 95% confidence interval (lower, upper)† based on validations of the fitted gradient boosted multiclass classifier (including age, sex and all eye movement variables) on Test-1 (N=133) and Test-2ǂ (N=244) datasets 25

Supplementary Table 9. Number of subjects for different levels of nicotine, caffeine and psychotropic medication and summary statistics (median, IQR) of HADS anxiety and HADS depression of different groups 27

Supplementary Table 10. Principal component regression models of the first 20 PC scores and different confounding variables (N=672) 28

Supplementary Table 11. Observed and predicted class and prediction probabilities of different classes of individuals in the test dataset (N=133) based on the fitted gradient boosted multiclass classifier (including sex and all eye movement features) 30

References 37

Machine learning workflow

We implemented a slightly modified version of a machine learning workflow of Maros et al[1] who provided a detailed account of the workflow in nested cross-validation (CV) framework, evaluation of the performance metrics and their assessments. The nested CV scheme works better when overfitting could be an issue with smaller sample sizes; the scheme separates the classifier learning problem from the calibration task and accurately estimates the external test error by averaging the performance metrics across folds. The 5x5 fold nested CV pipeline calibrates the classifier in the internal CV loop (innerfold) and accurately estimates the test error with the validation dataset in the external CV loop (outerfold), thus the calibration model only once uses the outerfold validation dataset for the prediction.

Briefly, the pipeline includes (see Figure 1): (1) partitioning the training dataset randomly into five sets, (2) including four sets as a calibration set and the remainder as a validation set (3) employing the selected classifier algorithm (gradient boosting) to conduct 5-fold cross-validation within the internal CV loop (S1.1; 1.2;…;1.5) and developing the tuned calibrator (M1.0), (4) fit the tuned calibrator to the validation dataset on the external CV loop to generate calibrated probabilities (P1.0), (5) repeating steps (2) to (4) for four remaining calibration and validation datasets and generate calibrated probabilities (P1.0; P2.0;…; P5.0), and (5) generating the final performance metrics (misclassification error, Brier score, log loss and AUC) based on the predictions on the external CV loops. The calibration methods used to obtain calibration probabilities were: Platt scaling for logistic regression[2] implemented in the R function *glm*, Firth's penalised logistic regression[3] implemented in the R package *brglm*[4] and ridge-penalised multinomial logistic regression[5] implemented in the R package *glmnet*. The optimal hyperparameters of the classifier were identified based on these performance metrics and the final model was developed using the full training data and five-fold repeated cross-validation.

The performance of the final model was then validated using two sets of testing data (Test-1, Test-2) with a range of performance metrics: area under the curve (AUC), sensitivity, specificity, positive predictive value (PPV), negative predictive value (NPV), F1 score, accuracy and balanced accuracy.

Three different estimates of AUC were obtained: the generalised overall AUC as proposed by Hand and Till[6], an overall groupwise AUC using one-versus-all (OVA) and pairwise AUCs using one-versus-one (OVO) methods. We conducted the bootstrap sampling with 1000 replicates to calculate the 95% confidence interval of these performance metrics.

We evaluated four classifiers (penalised multinomial logistic regression, support vector machine, random forest and gradient boosted decision tree). We found that the performance of the gradient boosted decision tree (GB) classifier was the best among all the four classifiers, therefore, this paper only presents the results of the GB classifier. The GB classifier is a representation of an ensemble of trees. We implemented the GB classifier using the extreme gradient boosting algorithm with softmax as the objective function for multiclass classification[7] and implemented in R package *xgboost*[8]. The GB model hyperparameters included: step size (*eta*), minimum loss reduction (*gamma*), maximum depth of a tree (*max_depth*), the minimum sum of child weight (*min_child_weight*), subsample ratio of the training instances (subsample), subsampling of columns (*colsample_bytree*), number of rounds for boosting (*nrounds*) and regularisation term on weights (*lambda*). All eye movement variables were pre-processed by centring and scaling each variable before fitting the GB model although this is not warranted in the context of the GB model. The hyperparameters of the final GB classifier were: *eta* (0.01), *gamma* (0.1), *max_depth* (6), *min_child_weight* (1), *subsample* (0.632), *colsample_bytree* (0.25), *nrounds* (5000) and *lambda* (1.00).

The complete pipeline was implemented in the R software environment[9] using appropriate packages. To execute the pipeline in a computationally efficient manner, we ran it on multi-core processors in a high-performance computing environment.

Table 1: Demographics of the patient and control subjects used for validating the classifier (Test-1 and Test-2).

	**Schizophrenia (SCZ)**	**Bipolar Affective Disorder (BPAD)**	**Major Depression Disorder (MDD)**	**Healthy Control (CON)**
**Validation data (Test-1; N=133)**				
N	30	35	33	35
Sex, Female:Male	5:25	20:15	18:15	21:14
Age (years), Median (Q1, Q3)	40.0 (32.0, 50.8)	51.0 (37.0, 56.5)	51.0 (33.0, 60.0)	30.0 (26.0, 47.5)
Education (years), Median (Q1, Q3)	13.0 (11.0, 13.5)	15.0 (13.4, 16.2)	12.0 (9.5, 13.0)	15.0 (15.0, 15.0)
Illness age of onset (years), Median (Q1, Q3)	24.0 (20.8, 27.5)	33.0 (24.0, 38.0)	23.5 (19.0, 35.0)	
Illness duration (years), Median (Q1, Q3)	19.0 (10.0, 24.0)	20.0 (13.0, 29.0)	11.0 (7.0, 23.0)	
CPZ, Median (Q1, Q3)	600.0 (300.0, 800.0)	50.0 (0.0, 200.0)	0.0 (0.0, 0.0)	
Nicotine (cigarettes/day), Median (Q1, Q3)	0.0 (0.0, 20.0)	0.0 (0.0, 8.0)	0.0 (0.0, 12.0)	0.0 (0.0, 0.0)
Caffeine intake (cups/day), Median (Q1, Q3)	3.5 (1.2, 5.0)	3.0 (1.0, 4.0)	5.0 (2.0, 6.0)	3.0 (1.0, 4.0)
HADS Anxiety, Median (Q1, Q3)	9.0 (5.8, 12.2)	8.0 (6.0, 10.0)	10.0 (6.0, 13.0)	3.5 (2.0, 6.0)
HADS Depression, Median (Q1, Q3)	7.0 (4.0, 10.0)	5.0 (2.8, 9.2)	8.0 (4.0, 10.0)	1.5 (0.0, 3.0)

	Schizophrenia (SCZ)	Bipolar Affective Disorder (BPAD)	Major Depression Disorder (MDD)	Healthy Control (CON)
**Validation data (Test-2; N=244)**				
N	60			184
Sex, Female:Male	25:35			105:79
Age (years), Median (Q1, Q3)	43.0 (37.8, 50.0)			29.5 (24.0, 47.2)
Education (years), Median (Q1, Q3)	13.5 (13.5, 15.0)			15.0 (14.0, 16.0)
Illness age of onset (years), Median (Q1, Q3)	26.5 (22.8, 31.0)			
Illness duration (years), Median (Q1, Q3)	12.0 (7.0, 21.0)			
CPZ, Median (Q1, Q3)	350.0 (125.5, 607.2)			
Nicotine (cigarettes/day), Median (Q1, Q3)	0.0 (0.0, 18.5)			0.0 (0.0, 0.0)
Caffeine intake (cups/day), Median (Q1, Q3)	2.0 (1.0, 4.0)			3.0 (2.0, 4.0)
HADS Anxiety, Median (Q1, Q3)	11.5 (8.0, 15.0)			4.0 (2.0, 7.0)
HADS Depression, Median (Q1, Q3)	NA (NA, NA)			1.0 (0.0, 3.0)

Q1, Q3 are first and third quartiles

Education indicates years in full time

CPZ = Chlorpromazine equivalents (mg/day) c)

Supplementary Table 2. Details of eye movement variables

SN	Variable name	Explanation
**Free viewing scanpath**		
1	FV_spath FNum_med	Free viewing saccade fixation frequency median U
2	FV_spath FDur_med	Free viewing saccade fixation duration median U
3	FV_spath SNum_med	Free viewing saccade frequency median U
4	FV_spath SDur_med	Free viewing saccade duration median U
5	FV_spath SAmp_med	Free viewing saccade amplitude median U
6	FV_spath SPVu_med	Free viewing saccade peak velocity median U
7	FV_spath SAVu_med	Free viewing saccade average velocity median U
8	FV_spath SLu_med	Free viewing saccade length median U
9	FV_Disp_med	Free viewing dispersion median U
**Horizontal smooth pursuit at 0.4Hz**		
10	SP_HS4_H_logSNR_med	Horizontal smooth pursuit log signal to noise ratio median
11	SP_HS4_H_RMSE_med	Horizontal smooth pursuit root mean square error median
12	SP_HS4_H_Gain_med	Horizontal smooth pursuit gain median
13	SP_spemHS4_FixNum5	Horizontal smooth pursuit fixation frequency median
14	SP_spemHS4_FixDur5	Horizontal smooth pursuit fixation duration median
15	SP_spemHS4_SacNum5	Horizontal smooth pursuit saccade frequency median
16	SP_spemHS4_SacDur5	Horizontal smooth pursuit saccade duration median
17	SP_spemHS4_SacAmp5	Horizontal smooth pursuit saccade amplitude median
18	SP_spemHS4_SacPV5	Horizontal smooth pursuit saccade peak velocity median
19	SP_spemHS4_SacAV5	Horizontal smooth pursuit saccade average velocity median

**Horizontal smooth pursuit at 0.6Hz**		
20	SP_HS6_H_logSNR_med	Horizontal smooth pursuit log signal to noise ratio median
21	SP_HS6_H_RMSE_med	Horizontal smooth pursuit root mean square error median
22	SP_HS6_H_Gain_med	Horizontal smooth pursuit gain median
23	SP_spemHS6_FixNum5	Horizontal smooth pursuit fixation frequency median
24	SP_spemHS6_FixDur5	Horizontal smooth pursuit fixation duration median
25	SP_spemHS6_SacNum5	Horizontal smooth pursuit saccade frequency median
26	SP_spemHS6_SacDur5	Horizontal smooth pursuit saccade duration median
27	SP_spemHS6_SacAmp5	Horizontal smooth pursuit saccade amplitude median
28	SP_spemHS6_SacPV5	Horizontal smooth pursuit saccade peak velocity median
29	SP_spemHS6_SacAV5	Horizontal smooth pursuit saccade average velocity median
**Lissajous smooth pursuit at 0.2Hz**		
30	SP_LS2_H_logSNR_med	Horizontal smooth pursuit log signal to noise ratio median
31	SP_LS2_H_RMSE_med	Horizontal smooth pursuit root mean square error median
32	SP_LS2_H_Gain_med	Horizontal smooth pursuit gain median
33	SP_LS2_V_logSNR_med	Vertical smooth pursuit log signal to noise ratio median
34	SP_LS2_V_RMSE_med	Vertical smooth pursuit root mean square error median
35	SP_LS2_V_Gain_med	Vertical smooth pursuit gain median
36	SP_spemLS2_FixNum5	Smooth pursuit fixation frequency median
37	SP_spemLS2_FixDur5	Smooth pursuit fixation duration median
38	SP_spemLS2_SacNum5	Smooth pursuit saccade frequency median
39	SP_spemLS2_SacDur5	Smooth pursuit saccade duration median
40	SP_spemLS2_SacAmp5	Smooth pursuit saccade amplitude median

41	SP_spemLS2_SacPV5	Smooth pursuit saccade peak velocity median
42	SP_spemLS2_SacAV5	Smooth pursuit saccade average velocity median
**Lissajous smooth pursuit at 0.4Hz**		
43	SP_LS4_H_logSNR_med	Horizontal smooth pursuit log signal to noise ratio median
44	SP_LS4_H_RMSE_med	Horizontal smooth pursuit root mean square error median
45	SP_LS4_H_Gain_med	Horizontal smooth pursuit gain median
46	SP_LS4_V_logSNR_med	Vertical smooth pursuit log signal to noise ratio median
47	SP_LS4_V_RMSE_med	Vertical smooth pursuit root mean square error median
48	SP_LS4_V_Gain_med	Vertical smooth pursuit gain median
49	SP_spemLS4_FixNum5	Smooth pursuit fixation frequency
50	SP_spemLS4_FixDur5	Smooth pursuit fixation duration
51	SP_spemLS4_SacNum5	Smooth pursuit saccade frequency
52	SP_spemLS4_SacDur5	Smooth pursuit saccade duration
53	SP_spemLS4_SacAmp5	Smooth pursuit saccade amplitude
54	SP_spemLS4_SacPV5	Smooth pursuit saccade peak velocity
55	SP_spemLS4_SacAV5	Smooth pursuit saccade average velocity
**Smooth pursuit at 0.4Hz with colour change**		
56	SP_HCX4_H_logSNR_med	Horizontal smooth pursuit log signal to noise ratio median
57	SP_HCX4_H_RMSE_med	Horizontal smooth pursuit root mean square error median
58	SP_HCX4_H_Gain_med	Horizontal smooth pursuit gain median
59	SP_spemHCX4_FixNum5	Smooth pursuit fixation frequency
60	SP_spemHCX4_FixDur5	Smooth pursuit fixation duration
61	SP_spemHCX4_SacNum5	Smooth pursuit saccade frequency
62	SP_spemHCX4_SacDur5	Smooth pursuit saccade duration

63	SP_spemHCX4_SacAmp5	Smooth pursuit saccade amplitude
64	SP_spemHCX4_SacPV5	Smooth pursuit saccade peak velocity
65	SP_spemHCX4_SacAV5	Smooth pursuit saccade average velocity
**Smooth pursuit at 0.4Hz with background task**		
66	SP_HS4B_H_logSNR_med	Horizontal smooth pursuit log signal to noise ratio median
67	SP_HS4B_H_RMSE_med	Horizontal smooth pursuit root mean square error median
68	SP_HS4B_H_Gain_med	Horizontal smooth pursuit gain median
69	SP_spemHS4B_FixNum5	Smooth pursuit fixation frequency
70	SP_spemHS4B_FixDur5	Smooth pursuit fixation duration
71	SP_spemHS4B_SacNum5	Smooth pursuit saccade frequency
72	SP_spemHS4B_SacDur5	Smooth pursuit saccade duration
73	SP_spemHS4B_SacAmp5	Smooth pursuit saccade amplitude
74	SP_spemHS4B_SacPV5	Smooth pursuit saccade peak velocity
75	SP_spemHS4B_SacAV5	Smooth pursuit saccade average velocity
**Smooth pursuit Lissajous at 0.4Hz with background task**		
76	SP_LS2B_H_logSNR_med	Horizontal smooth pursuit log signal to noise ratio median
77	SP_LS2B_H_RMSE_med	Horizontal smooth pursuit root mean square error median
78	SP_LS2B_H_Gain_med	Horizontal smooth pursuit gain median
79	SP_LS2B_V_logSNR_med	Vertical smooth pursuit log signal to noise ratio median
80	SP_LS2B_V_RMSE_med	Vertical smooth pursuit root mean square error median
81	SP_LS2B_V_Gain_med	Vertical smooth pursuit gain median
82	SP_spemLS2B_FixNum5	Smooth pursuit fixation frequency
83	SP_spemLS2B_FixDur5	Smooth pursuit fixation duration
84	SP_spemLS2B_SacNum5	Smooth pursuit saccade frequency

85	SP_spemLS2B_SacDur5	Smooth pursuit saccade duration
86	SP_spemLS2B_SacAmp5	Smooth pursuit saccade amplitude
87	SP_spemLS2B_SacPV5	Smooth pursuit saccade peak velocity
88	SP_spemLS2B_SacAV5	Smooth pursuit saccade average velocity
**Fixation maintenance task**		
89	FIX_FS_FN	Fixation frequency
90	FIX_FS_FD	Fixation duration
91	FIX_FS_SN	Saccade frequency
92	FIX_FS_SA	Saccade amplitude
93	FIX_FS_SPL	Saccade length
94	FIX_FD_FN	Fixation frequency
95	FIX_FD_FD	Fixation duration
96	FIX_FD_SN	Saccade frequency
97	FIX_FD_SA	Saccade amplitude
98	FIX_FD_SPL	Saccade length

Supplementary Table 3. Summary statistics of eye movement variables used in the study (N=672)

For the description of eye movement variables, see Supplementary Table 2

SN	Variables	Median	Min	Max	Q1	Q3
1	FV_spathFNum_med	24.70	6.50	34.00	22.00	27.00
2	FV_spathFDur_med	250.03	131.30	1255.40	224.62	281.29
3	FV_spathSNum_med	25.00	10.68	37.00	22.00	27.15
4	FV_spathSDur_med	36.87	13.50	163.00	32.30	42.78
5	FV_spathSAmp_med	3.77	0.55	7.36	3.07	4.41
6	FV_spathSPVu_med	233.65	11.00	957.55	204.38	276.00
7	FV_spathSAVu_med	101.57	4.54	173.46	87.92	115.18
8	FV_spathSLu_med	759.24	55.45	1396.46	599.32	891.01
9	FV_Disp_med	0.82	0.00	1.60	0.68	0.98
10	SP_HS4_H_logSNR_med	1.89	0.12	5.29	1.65	2.44
11	SP_HS4_H_RMSE_med	35.06	11.33	435.81	23.76	64.78
12	SP_HS4_H_Gain_med	0.99	0.73	1.07	0.99	1.00
13	SP_spemHS4_FixNum5	44.00	17.00	80.00	38.00	52.00
14	SP_spemHS4_FixDur5	290.50	103.00	902.00	248.00	349.75
15	SP_spemHS4_SacNum5	46.00	17.00	121.00	38.75	55.00
16	SP_spemHS4_SacDur5	26.00	9.00	113.00	20.00	33.04
17	SP_spemHS4_SacAmp5	1.41	0.51	7.88	1.10	1.91
18	SP_spemHS4_SacPV5	128.00	18.00	439.00	98.50	163.50
19	SP_spemHS4_SacAV5	56.74	13.92	151.72	46.94	69.13
20	SP_HS6_H_logSNR_med	1.73	-0.09	3.65	1.49	2.23

21	SP_HS6_H_RMSE_med	57.03	19.18	430.11	38.06	82.28
22	SP_HS6_H_Gain_med	1.01	0.99	1.15	1.01	1.01
23	SP_spemHS6_FixNum5	53.00	12.00	78.00	46.00	59.12
24	SP_spemHS6_FixDur5	240.00	108.50	710.00	210.75	278.96
25	SP_spemHS6_SacNum5	57.00	24.00	130.00	50.00	65.00
26	SP_spemHS6_SacDur5	30.00	13.00	133.00	24.00	37.00
27	SP_spemHS6_SacAmp5	2.17	0.64	12.53	1.68	2.78
28	SP_spemHS6_SacPV5	169.25	44.00	487.00	130.01	216.00
29	SP_spemHS6_SacAV5	70.33	20.79	169.07	59.57	84.41
30	SP_LS2_H_logSNR_med	1.70	0.04	4.70	1.50	2.28
31	SP_LS2_H_RMSE_med	21.33	6.21	238.24	15.38	36.97
32	SP_LS2_H_Gain_med	1.10	0.90	1.39	1.09	1.12
33	SP_LS2_V_logSNR_med	1.62	-0.77	5.31	1.37	2.26
34	SP_LS2_V_RMSE_med	30.59	8.77	456.15	21.00	46.89
35	SP_LS2_V_Gain_med	0.92	0.75	1.16	0.90	0.96
36	SP_spemLS2_FixNum5	39.00	9.00	87.00	31.00	46.00
37	SP_spemLS2_FixDur5	335.50	108.00	1956.00	278.00	413.00
38	SP_spemLS2_SacNum5	40.00	9.00	90.00	33.00	49.00
39	SP_spemLS2_SacDur5	22.00	8.00	190.00	18.00	27.00
40	SP_spemLS2_SacAmp5	0.98	0.34	4.94	0.79	1.24
41	SP_spemLS2_SacPV5	89.00	39.00	318.00	73.00	111.12
42	SP_spemLS2_SacAV5	46.70	10.42	125.22	40.56	53.05
43	SP_LS4_H_logSNR_med	1.71	-0.74	5.16	1.54	2.34
44	SP_LS4_H_RMSE_med	28.78	11.14	280.41	22.65	44.80

45	SP_LS4_H_Gain_med	1.01	0.89	1.10	1.00	1.02
46	SP_LS4_V_logSNR_med	1.82	-0.58	4.74	1.58	2.33
47	SP_LS4_V_RMSE_med	44.01	13.27	390.63	33.64	64.98
48	SP_LS4_V_Gain_med	0.99	0.88	1.15	0.98	1.00
49	SP_spemLS4_FixNum5	53.00	23.00	89.00	46.00	59.00
50	SP_spemLS4_FixDur5	258.25	116.00	512.00	223.79	292.00
51	SP_spemLS4_SacNum5	56.00	27.00	123.00	49.00	63.34
52	SP_spemLS4_SacDur5	28.00	10.00	104.00	23.46	34.00
53	SP_spemLS4_SacAmp5	1.82	0.40	8.81	1.48	2.28
54	SP_spemLS4_SacPV5	134.00	49.00	354.50	109.88	173.72
55	SP_spemLS4_SacAV5	64.29	16.74	145.21	56.86	72.72
56	SP_HCX4_H_logSNR_med	1.96	0.00	5.44	1.80	2.55
57	SP_HCX4_H_RMSE_med	28.44	9.97	300.09	21.38	43.33
58	SP_HCX4_H_Gain_med	0.99	0.94	1.05	0.99	1.00
59	SP_spemHCX4_FixNum5	42.00	1.00	78.00	35.00	50.00
60	SP_spemHCX4_FixDur5	306.75	98.00	772.00	259.88	369.62
61	SP_spemHCX4_SacNum5	43.00	11.00	104.00	36.00	53.00
62	SP_spemHCX4_SacDur5	24.00	10.00	908.00	20.00	30.00
63	SP_spemHCX4_SacAmp5	1.18	0.42	7.18	0.95	1.48
64	SP_spemHCX4_SacPV5	113.00	0.00	638.00	93.00	138.12
65	SP_spemHCX4_SacAV5	51.22	8.35	131.81	41.41	60.77
66	SP_HS4B_H_logSNR_med	1.87	-0.15	4.04	1.56	2.28
67	SP_HS4B_H_RMSE_med	46.64	10.27	273.76	29.77	75.14
68	SP_HS4B_H_Gain_med	0.99	0.93	1.08	0.99	1.00

69	SP_spemHS4B_FixNum5	47.00	12.00	79.00	40.70	54.00
70	SP_spemHS4B_FixDur5	280.00	128.00	838.00	237.00	328.00
71	SP_spemHS4B_SacNum5	49.00	18.00	119.00	42.00	58.00
72	SP_spemHS4B_SacDur5	26.00	9.00	162.00	22.00	34.00
73	SP_spemHS4B_SacAmp5	1.62	0.38	6.31	1.25	2.19
74	SP_spemHS4B_SacPV5	137.75	52.00	416.00	110.38	182.62
75	SP_spemHS4B_SacAV5	61.24	18.79	110.43	51.11	75.11
76	SP_LS2B_H_logSNR_med	1.69	0.32	4.34	1.48	2.23
77	SP_LS2B_H_RMSE_med	26.62	6.45	149.63	18.73	42.65
78	SP_LS2B_H_Gain_med	1.10	0.92	1.37	1.09	1.12
79	SP_LS2B_V_logSNR_med	1.65	-2.65	3.69	1.42	2.18
80	SP_LS2B_V_RMSE_med	35.51	8.76	257.38	23.55	50.72
81	SP_LS2B_V_Gain_med	0.92	0.77	1.11	0.90	0.95
82	SP_spemLS2B_FixNum5	47.00	14.00	74.00	39.98	54.00
83	SP_spemLS2B_FixDur5	297.00	137.00	756.00	253.00	357.00
84	SP_spemLS2B_SacNum5	49.00	14.00	82.00	41.00	56.00
85	SP_spemLS2B_SacDur5	24.00	10.00	88.00	20.00	30.00
86	SP_spemLS2B_SacAmp5	1.31	0.40	3.21	1.04	1.63
87	SP_spemLS2B_SacPV5	108.00	48.50	296.00	90.75	136.12
88	SP_spemLS2B_SacAV5	54.30	11.10	102.42	47.22	61.89
89	FIX_FS_FN	5.43	0.50	499.43	3.43	7.57
90	FIX_FS_FD	922.12	0.40	5426.00	502.57	1871.86
91	FIX_FS_SN	4.71	0.00	174.71	2.50	7.50
92	FIX_FS_SA	0.58	0.00	1363.00	0.36	0.87

93	FIX_FS_SPL	19.16	0.00	941.64	7.82	37.02
94	FIX_FD_FN	5.50	1.00	520.09	3.50	8.25
95	FIX_FD_FD	884.20	0.50	21857.67	491.50	1825.27
96	FIX_FD_SN	4.90	0.00	83.18	2.75	8.00
97	FIX_FD_SA	0.65	0.00	1363.00	0.44	1.00
98	FIX_FD_SPL	22.09	0.00	1631.01	9.38	45.47

Supplementary Figure 1. Correlation plot of all eye movement variables (N=672)

For the description of eye movement variables, see Supplementary Table 1

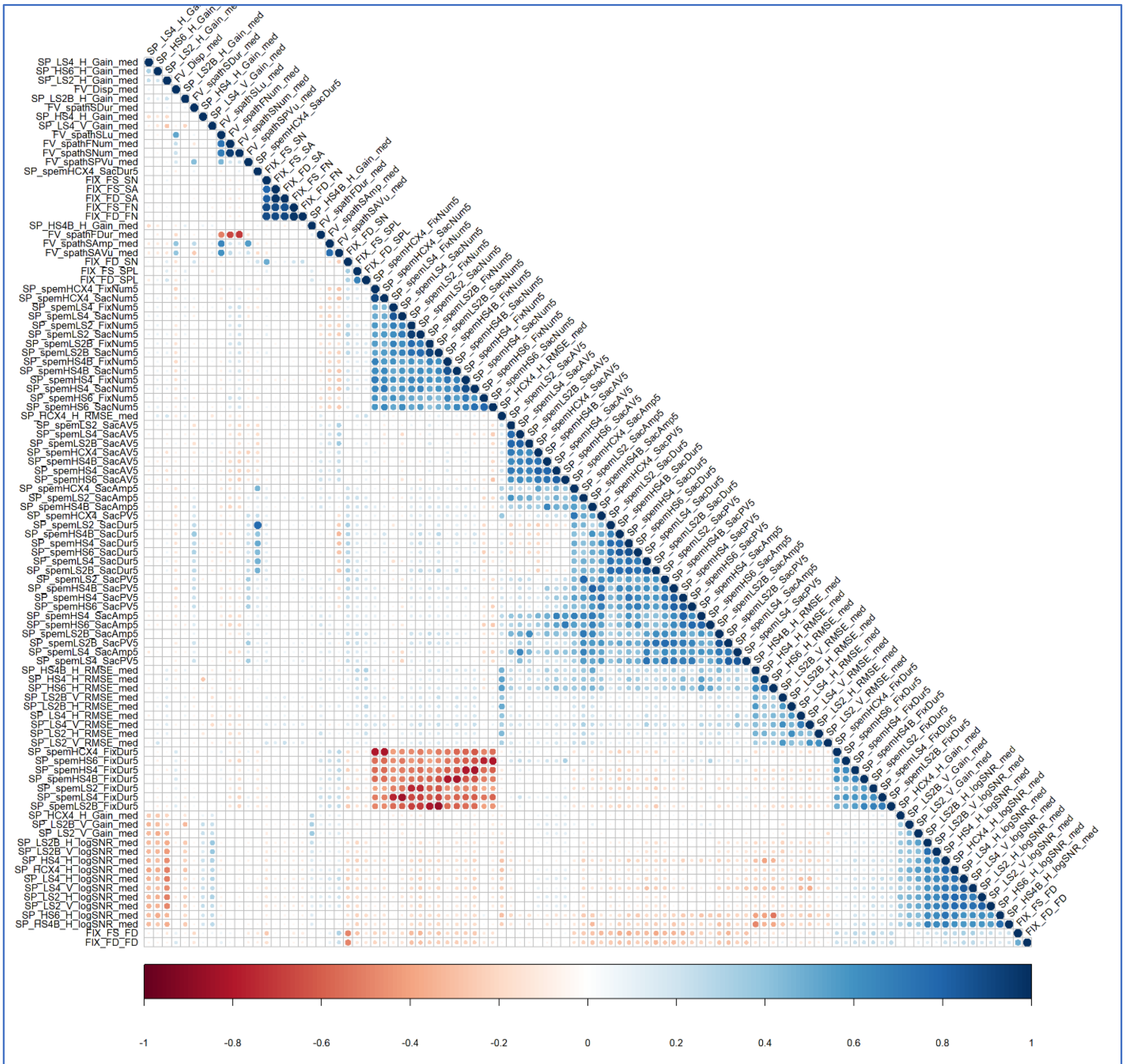

Supplementary Table 4. Detailed performance metrics along with the 95% confidence interval (lower, upper)† based on validations of the fitted gradient boosted multiclass classifier (including all eye movement features and sex) on Test-1 (N=133) and Test-2ǂ (N=244) datasets

The table provides further details on performance metrics provided in Table 3

Statistics	CON	SCZ	BPAD	MDD
**Validation on Test-1 (N=133)**				
AUC (OVA)§	0.85 (0.74, 0.90)	0.85 (0.72, 0.88)	0.78 (0.62, 0.83)	0.76 (0.60, 0.83)
AUC (OVO)§ with CON		0.84 (0.70, 0.90)	0.85 (0.74, 0.91)	0.82 (0.68, 0.88)
AUC (OVO)§ with SCZ			0.82 (0.65, 0.86)	0.84 (0.71, 0.89)
AUC (OVO)§ with BPAD				0.69 (0.50, 0.77)
Overall AUC§§		0.81 (0.71, 0.83)		
Sensitivity	0.57 (0.31, 0.69)	0.60 (0.3, 0.73)	0.49 (0.23, 0.66)	0.55 (0.27, 0.70)
Specificity	0.86 (0.79, 0.95)	0.85 (0.77, 0.92)	0.87 (0.72, 0.91)	0.82 (0.68, 0.88)
Positive Predictive Value (PPV)	0.59 (0.41, 0.77)	0.55 (0.35, 0.67)	0.57 (0.31, 0.67)	0.50 (0.28, 0.59)
Negative Predictive Value (NPV)	0.85 (0.78, 0.89)	0.88 (0.81, 0.92)	0.83 (0.75, 0.87)	0.85 (0.76, 0.89)
F1 Score	0.58 (0.37, 0.7)	0.57 (0.34, 0.67)	0.52 (0.28, 0.63)	0.52 (0.28, 0.62)
Balanced Accuracy	0.74 (0.58, 0.79)	0.77 (0.58, 0.79)	0.74 (0.53, 0.75)	0.75 (0.52, 0.75)

Statistics	CON	SCZ	BPAD	MDD
**Validation on Test-2 (N=244)**				
AUC (OVA)	0.64 (0.51, 0.74)	0.89 (0.78, 0.93)		
AUC (OVO) with CON		0.77 (0.65, 0.83)		
Sensitivity	0.63 (0.52, 0.7)	0.58 (0.35, 0.72)		
Specificity	0.60 (0.40, 0.75)	0.91 (0.87, 0.96)		
Positive Predictive Value (PPV)	0.83 (0.75, 0.88)	0.67 (0.54, 0.83)		
Negative Predictive Value (NPV)	0.34 (0.24, 0.41)	0.87 (0.81, 0.91)		
F1 Score	0.71 (0.62, 0.76)	0.63 (0.44, 0.73)		
Balanced Accuracy	0.61 (0.49, 0.69)	0.75 (0.63, 0.81)		

[†]The bootstrap sampling with 1000 replicates from the relevant datasets was used to calculate the 95% confidence interval of different performance metrics.

[‡]The validation on Test-2 dataset does not have any representation of BPAD and MDD patients.

[§]AUC (area under the curve) represents the overall groupwise AUC by one-versus-all (OVA) and pairwise AUCs by one-versus-one (OVO) methods.

[§§]The overall AUC is the generalised overall AUC as proposed by Hand and Till (2001).

CON: Healthy Control; SCZ: Schizophrenia; BPAD: Bipolar Affective Disorder; MDD: Major Depression Disorder

Supplementary Table 5. Variable importance of the fitted gradient boosted multiclass classifier (including all eye movement features and sex) and corresponding median (Q1, Q3) of all eye movement variables in Test 1 data (N=133).

For the description of eye movement variables, see Supplementary Table 2

SN	Eye variables	Var Imp (%)	Plot	CON	SCZ	BPAD	MDD
1	FV_Disp_med	100.00		0.92 (0.79, 0.99)	0.75 (0.56, 0.85)	0.79 (0.70, 0.93)	0.81 (0.69, 0.89)
2	FV_spathSAVu_med	94.77		111.14 (99.43, 118.79)	98.04 (82.54, 105.11)	97.61 (86.81, 112.27)	96.70 (75.72, 115.21)
3	SP_LS4_H_logSNR_med	85.48		2.23 (1.71, 2.53)	1.90 (1.42, 2.36)	1.61 (1.56, 2.04)	1.66 (1.46, 1.79)
4	FV_spathSLu_med	84.02		903.92 (739.50, 979.86)	667.66 (506.56, 752.29)	783.18 (609.70, 889.60)	803.65 (636.61, 1009.73)
5	SP_HS6_H_Gain_med	81.50		1.010 (1.006, 1.014)	1.009 (1.005, 1.013)	1.013 (1.010, 1.014)	1.013 (1.010, 1.014)
6	SP_LS2B_H_RMSE_med	75.60		26.84 (20.04, 38.07)	31.57 (19.77, 46.88)	28.08 (17.78, 38.14)	31.65 (22.99, 49.40)
7	FIX_FD_SPL	75.17		14.77 (8.65, 25.98)	25.82 (15.31, 92.44)	28.79 (13.79, 98.60)	23.00 (12.27, 28.93)
8	SP_LS2_H_Gain_med	69.45		1.094 (1.040, 1.109)	1.094 (1.052, 1.117)	1.108 (1.097, 1.120)	1.103 (1.095, 1.114)
9	SP_HCX4_H_logSNR_med	67.39		2.48 (1.85, 2.67)	2.15 (1.87, 3.12)	1.90 (1.72, 2.43)	1.89 (1.75, 2.33)
10	FV_spathSDur_med	65.33		34.31 (31.25, 38.75)	35.94 (30.00, 41.69)	40.00 (34.00, 43.15)	39.90 (36.75, 44.05)
11	FIX_FD_SN	63.77		4.09 (1.98, 6.00)	5.42 (3.75, 10.61)	7.36 (4.38, 10.10)	4.89 (3.50, 6.73)
12	SP_spemLS2B_SacAV5	55.98		53.78 (49.20, 57.38)	57.32 (51.27, 65.96)	55.80 (46.55, 60.62)	52.71 (45.62, 59.50)
13	SP_LS2B_V_Gain_med	55.05		0.944 (0.897, 0.952)	0.936 (0.900, 0.960)	0.918 (0.890, 0.950)	0.899 (0.893, 0.946)
14	FV_spathFNum_med	51.17		26.00 (22.82, 28.29)	24.23 (22.25, 25.57)	25.00 (23.70, 26.48)	26.07 (24.57, 27.64)
15	SP_LS2_V_Gain_med	46.61		0.946 (0.915, 0.980)	0.938 (0.908, 0.970)	0.909 (0.896, 0.948)	0.908 (0.901, 0.933)
16	SP_LS2_H_RMSE_med	46.00		19.18 (12.69, 36.78)	22.32 (15.17, 31.20)	19.75 (16.30, 28.58)	20.53 (16.67, 42.35)
17	SP_spemHS4B_SacAV5	46.00		65.00 (55.97, 78.95)	73.23 (58.83, 76.68)	59.52 (50.63, 72.55)	56.21 (46.22, 63.81)
18	SP_spemHCX4_SacAV5	45.57		55.25 (47.52, 61.25)	59.12 (47.38, 67.51)	47.99 (42.81, 55.26)	48.75 (38.94, 54.29)
19	FIX_FS_FD	45.16		1047.43 (590.61, 1888.00)	944.39 (344.50, 1557.70)	534.50 (390.88, 1030.27)	937.71 (568.00, 1774.50)
20	FV_spathFDur_med	44.08		246.00 (216.50, 275.25)	254.33 (231.61, 291.62)	246.00 (226.00, 263.80)	229.50 (206.00, 261.36)
21	SP_LS4_V_logSNR_med	43.14		2.21 (1.69, 2.50)	2.02 (1.49, 2.45)	1.78 (1.64, 2.06)	1.73 (1.50, 1.90)
22	FV_spathSPVu_med	42.70		220.75 (203.96, 254.38)	219.09 (190.01, 260.73)	243.50 (207.25, 286.71)	259.99 (239.46, 301.65)
23	SP_HCX4_H_RMSE_med	42.40		24.03 (19.80, 34.14)	30.51 (24.22, 37.79)	28.22 (22.68, 52.65)	35.22 (22.18, 63.68)
24	FIX_FD_FD	40.84		1100.45 (656.76, 2213.97)	672.05 (391.32, 1149.13)	558.09 (364.74, 972.28)	759.00 (515.00, 1427.62)
25	FIX_FD_FN	39.88		5.00 (2.89, 6.60)	6.07 (4.75, 11.00)	7.82 (5.02, 10.74)	5.73 (3.75, 7.25)

Supplementary Table 5 (continued)

26	SP_HCX4_H_Gain_med	39.81		0.994 (0.987, 0.998)	0.992 (0.988, 0.999)	0.991 (0.986, 0.997)	0.987 (0.983, 0.993)
27	SP_LS2B_H_Gain_med	39.55		1.104 (1.097, 1.121)	1.095 (1.085, 1.110)	1.105 (1.093, 1.120)	1.101 (1.097, 1.112)
28	FIX_FS_SPL	38.92		13.26 (7.54, 30.99)	17.98 (8.97, 58.12)	30.49 (13.00, 60.28)	14.61 (6.09, 28.95)
29	FV_spathSAmp_med	37.21		4.13 (3.46, 4.37)	3.31 (2.92, 3.90)	3.57 (3.07, 4.45)	3.95 (2.94, 4.74)
30	SP_HS6_H_RMSE_med	36.44		46.16 (36.70, 66.54)	56.25 (45.67, 83.72)	72.35 (47.51, 104.68)	60.38 (35.61, 96.82)
31	SP_LS2B_V_logSNR_med	35.10		2.21 (1.66, 2.35)	1.83 (1.47, 2.21)	1.61 (1.46, 1.93)	1.49 (1.39, 1.61)
32	SP_LS2_V_RMSE_med	33.96		25.82 (19.46, 38.59)	28.70 (21.60, 32.48)	31.83 (24.02, 42.35)	33.34 (23.50, 55.99)
33	SP_HS4B_H_Gain_med	33.93		0.994 (0.989, 0.998)	0.990 (0.984, 0.996)	0.988 (0.984, 0.994)	0.988 (0.983, 0.994)
34	SP_LS2_V_logSNR_med	33.27		2.05 (1.51, 2.49)	1.80 (1.43, 2.65)	1.60 (1.41, 1.98)	1.52 (1.39, 1.69)
35	SP_spemHS4B_SacDur5	32.85		24.00 (21.58, 28.00)	28.00 (23.41, 37.50)	32.00 (25.50, 36.00)	31.00 (25.19, 38.00)
36	SP_HS4_H_logSNR_med	32.34		2.28 (1.83, 2.60)	2.13 (1.85, 2.68)	1.76 (1.65, 1.97)	1.84 (1.55, 2.04)
37	FIX_FD_SA	32.28		0.57 (0.33, 0.76)	1.00 (0.51, 1.69)	0.83 (0.47, 1.13)	0.56 (0.42, 0.87)
38	FV_spathSNum_med	31.36		26.00 (23.06, 29.00)	24.29 (21.38, 26.78)	25.50 (23.50, 27.00)	27.31 (24.73, 28.79)
39	SP_LS2_H_logSNR_med	29.73		1.96 (1.65, 2.50)	1.95 (1.58, 3.15)	1.60 (1.41, 2.06)	1.70 (1.50, 2.05)
40	SP_LS4_H_Gain_med	29.59		1.010 (1.004, 1.014)	1.008 (0.999, 1.014)	1.013 (1.008, 1.017)	1.015 (1.008, 1.017)
41	SP_spemHCX4_FixDur5	28.70		310.00 (267.00, 373.00)	299.25 (263.50, 361.00)	282.00 (232.50, 343.00)	290.50 (250.00, 381.00)
42	SP_spemLS2B_FixDur5	28.39		293.00 (252.53, 358.80)	282.00 (242.45, 364.59)	292.00 (239.50, 320.00)	286.00 (244.00, 313.00)
43	SP_HS4_H_RMSE_med	28.06		28.76 (23.90, 54.80)	37.07 (24.93, 63.39)	45.20 (27.93, 74.03)	38.57 (24.23, 71.59)
44	SP_LS4_V_RMSE_med	26.96		42.44 (33.82, 50.61)	49.29 (33.89, 78.39)	48.66 (38.90, 67.82)	44.36 (35.27, 65.29)
45	SP_spemHS4_SacAV5	26.18		58.93 (49.67, 71.38)	64.38 (51.39, 75.52)	57.00 (49.06, 66.45)	50.55 (42.89, 59.09)
46	SP_spemHCX4_SacPV5	25.56		99.00 (88.75, 121.74)	121.75 (111.88, 145.88)	125.50 (102.75, 156.00)	109.50 (87.50, 132.50)
47	SP_HS4_H_Gain_med	25.29		0.994 (0.988, 1.000)	0.991 (0.985, 1.001)	0.990 (0.987, 0.996)	0.987 (0.984, 0.992)
48	SP_HS4B_H_RMSE_med	25.24		39.91 (30.81, 61.43)	47.49 (36.74, 87.35)	55.86 (39.38, 82.95)	54.32 (40.50, 85.64)
49	SP_LS2B_H_logSNR_med	24.93		2.17 (1.63, 2.29)	1.79 (1.57, 2.24)	1.67 (1.43, 1.98)	1.60 (1.36, 1.96)
50	SP_spemLS4_SacAmp5	24.73		1.75 (1.37, 2.15)	2.04 (1.83, 2.54)	2.13 (1.81, 2.37)	1.98 (1.67, 2.35)

Supplementary Table 5 (continued)

51	SP_spemHS4B_FixDur5	24.28		293.33 (258.00, 337.51)	273.15 (238.12, 313.25)	251.00 (217.66, 293.00)	251.00 (228.00, 322.00)
52	FIX_FS_SA	23.80		0.58 (0.36, 0.88)	0.55 (0.34, 0.96)	0.68 (0.38, 0.95)	0.54 (0.40, 0.74)
53	SP_spemHS6_SacNum5	23.78		56.00 (50.50, 63.57)	61.00 (51.65, 70.50)	57.00 (48.50, 64.00)	60.00 (54.00, 74.00)
54	SP_HS4B_H_logSNR_med	23.37		2.11 (1.60, 2.44)	1.93 (1.63, 2.36)	1.65 (1.48, 1.90)	1.68 (1.32, 1.95)
55	SP_spemLS4_SacAV5	23.08		67.50 (58.83, 71.87)	66.31 (60.68, 74.43)	64.78 (59.36, 71.75)	62.00 (55.00, 69.53)
56	SP_spemLS2_SacAV5	22.58		44.86 (41.33, 49.25)	49.22 (42.47, 52.76)	48.02 (44.14, 50.88)	44.00 (37.43, 52.07)
57	SP_LS4_H_RMSE_med	22.41		26.58 (23.23, 37.72)	29.35 (20.94, 47.11)	29.29 (25.58, 43.49)	32.41 (23.53, 48.96)
58	SP_HS6_H_logSNR_med	21.36		2.06 (1.74, 2.38)	1.95 (1.67, 2.35)	1.55 (1.20, 1.72)	1.61 (1.31, 1.81)
59	SP_spemHS6_SacAV5	20.87		76.45 (67.22, 88.19)	73.95 (61.29, 86.64)	70.00 (59.56, 84.90)	64.97 (55.53, 76.78)
60	SP_LS2B_V_RMSE_med	20.69		29.92 (22.15, 55.16)	40.44 (26.29, 46.86)	34.62 (26.46, 45.36)	41.44 (26.56, 52.16)
61	SP_LS4_V_Gain_med	20.52		0.993 (0.984, 1.000)	0.988 (0.983, 0.997)	0.986 (0.984, 0.996)	0.989 (0.985, 0.994)
62	SP_spemLS4_FixNum5	20.26		54.00 (48.50, 58.14)	52.96 (41.00, 60.90)	54.00 (45.10, 62.50)	55.29 (52.78, 59.85)
63	SP_spemLS4_SacPV5	19.81		123.00 (101.00, 145.00)	142.25 (123.88, 201.18)	155.12 (135.52, 204.04)	150.50 (120.00, 182.00)
64	SP_spemHCX4_SacDur5	19.79		20.00 (18.25, 24.00)	23.50 (20.00, 28.00)	29.00 (22.00, 32.00)	27.50 (21.00, 31.00)
65	SP_spemHS6_SacDur5	18.65		27.86 (24.99, 29.46)	29.75 (25.93, 35.75)	34.00 (28.00, 40.00)	33.50 (25.00, 40.00)
66	FIX_FS_FN	18.38		5.00 (3.00, 6.07)	6.29 (4.00, 9.96)	7.57 (4.68, 9.00)	5.70 (3.50, 8.00)
67	SP_spemLS2_SacAmp5	18.24		0.82 (0.71, 1.13)	1.10 (0.97, 1.30)	1.08 (0.93, 1.36)	0.99 (0.85, 1.21)
68	SP_spemHS4B_SacNum5	18.22		48.00 (43.00, 57.80)	49.50 (46.02, 60.10)	55.00 (46.50, 61.29)	52.00 (44.00, 62.00)
69	FIX_FS_SN	18.18		4.14 (2.07, 5.46)	5.71 (3.00, 9.32)	7.14 (4.39, 8.96)	5.00 (2.50, 7.00)
70	SP_spemLS2B_SacAmp5	17.66		1.22 (0.98, 1.34)	1.47 (1.23, 1.93)	1.47 (1.22, 1.79)	1.39 (1.23, 1.69)
71	SP_spemHCX4_FixNum5	17.60		40.00 (35.50, 47.00)	42.00 (35.25, 51.75)	47.00 (40.00, 57.00)	40.00 (33.00, 56.00)
72	SP_spemLS2_SacPV5	17.29		73.00 (63.25, 96.25)	95.50 (81.50, 119.62)	100.50 (87.00, 117.25)	91.00 (75.00, 112.00)
73	SP_spemHS6_FixNum5	17.27		53.00 (47.82, 60.50)	54.97 (48.79, 59.75)	52.00 (45.50, 59.00)	55.00 (49.00, 66.00)
74	SP_spemLS2B_SacPV5	16.59		98.50 (85.52, 109.10)	119.82 (97.75, 155.38)	123.00 (103.25, 147.50)	126.00 (103.50, 154.00)
75	SP_spemHS4B_SacAmp5	16.29		1.50 (1.24, 1.94)	1.95 (1.51, 2.38)	1.86 (1.43, 2.35)	1.52 (1.26, 2.25)

Supplementary Table 5 (continued)

76	SP_spemHS6_SacPV5	16.27		154.50 (131.50, 193.64)	183.29 (135.75, 230.38)	196.00 (149.25, 241.25)	176.00 (135.00, 208.00)
77	SP_spemLS2_FixDur5	15.86		330.00 (278.25, 367.00)	322.00 (261.08, 463.25)	327.50 (252.50, 390.00)	321.50 (285.50, 359.50)
78	SP_spemHS4B_FixNum5	15.03		43.73 (41.00, 53.40)	47.00 (44.00, 55.92)	51.00 (46.00, 56.81)	49.00 (41.00, 58.00)
79	SP_spemHS4B_SacPV5	15.03		126.00 (111.47, 152.61)	153.25 (127.95, 195.75)	166.00 (124.50, 209.75)	148.00 (112.00, 170.00)
80	SP_spemLS4_FixDur5	14.78		260.00 (222.00, 280.00)	253.50 (208.25, 295.00)	259.00 (211.25, 282.25)	245.00 (216.00, 268.85)
81	SP_spemHS4_SacPV5	13.36		111.00 (97.50, 138.50)	133.50 (114.00, 169.75)	142.00 (110.50, 189.00)	128.00 (97.00, 149.00)
82	SP_spemHS6_FixDur5	13.08		244.00 (224.96, 272.13)	227.50 (205.12, 250.25)	223.00 (204.00, 278.25)	223.00 (183.00, 264.00)
83	SP_spemHS6_SacAmp5	12.84		2.09 (1.80, 2.53)	2.44 (1.87, 2.85)	2.39 (1.91, 3.06)	2.07 (1.76, 2.50)
84	Sex	12.24					
85	SP_spemHCX4_SacAmp5	11.66		1.06 (0.94, 1.22)	1.35 (1.12, 1.73)	1.36 (1.10, 1.52)	1.09 (0.91, 1.48)
86	SP_spemHS4_FixDur5	11.66		304.00 (260.25, 350.50)	282.00 (229.75, 356.75)	260.00 (218.50, 311.50)	280.00 (247.00, 317.00)
87	SP_spemLS2B_FixNum5	10.69		49.00 (40.34, 54.00)	47.00 (39.02, 52.94)	50.00 (46.50, 56.00)	50.00 (46.00, 55.00)
88	SP_spemLS2B_SacDur5	10.62		21.71 (20.00, 24.68)	24.00 (21.99, 30.65)	26.00 (23.00, 32.50)	27.00 (22.95, 34.00)
89	SP_spemHS4_SacAmp5	9.76		1.30 (1.11, 1.77)	1.72 (1.31, 1.91)	1.52 (1.35, 1.98)	1.25 (1.08, 1.73)
90	SP_spemLS2_SacNum5	8.94		42.00 (36.50, 51.50)	43.50 (30.00, 51.50)	44.00 (35.50, 55.50)	43.00 (38.00, 50.00)
91	SP_spemLS2B_SacNum5	8.13		51.00 (41.65, 56.50)	51.00 (41.29, 55.75)	51.00 (47.00, 59.50)	51.00 (46.00, 59.00)
92	SP_spemLS2_FixNum5	7.57		42.00 (35.00, 46.50)	39.50 (28.25, 48.75)	42.00 (35.00, 50.50)	42.00 (36.00, 46.00)
93	SP_spemHS4_SacDur5	7.44		24.00 (20.00, 28.00)	24.00 (20.00, 30.75)	27.00 (24.00, 34.25)	29.00 (22.00, 34.00)
94	SP_spemHCX4_SacNum5	6.77		40.00 (37.00, 50.00)	43.50 (35.75, 53.50)	48.00 (41.00, 58.50)	41.00 (33.00, 59.00)
95	SP_spemLS4_SacNum5	6.66		56.00 (53.00, 63.50)	55.37 (48.50, 66.00)	56.00 (48.00, 66.50)	60.00 (55.00, 65.00)
96	SP_spemLS4_SacDur5	6.55		25.00 (24.00, 29.71)	28.53 (25.19, 37.36)	33.00 (26.50, 36.33)	31.00 (26.00, 38.00)
97	SP_spemLS2_SacDur5	5.82		19.00 (16.00, 23.00)	22.00 (18.12, 25.17)	24.00 (21.75, 29.00)	23.00 (19.50, 30.00)
98	SP_spemHS4_SacNum5	4.44		45.00 (39.50, 52.50)	50.00 (40.50, 58.75)	52.00 (46.50, 57.50)	48.00 (42.00, 59.00)
99	SP_spemHS4_FixNum5	0.00		43.00 (38.50, 49.50)	45.50 (35.50, 54.50)	49.00 (42.00, 57.00)	47.00 (41.00, 56.00)

CON: Healthy Control; SCZ: Schizophrenia; BPAD: Bipolar Affective Disorder; MDD: Major Depression Disorder

For some eye movement variables with small values, summary statistics are presented up to three decimal places.

Supplementary Table 6. Confusion matrix and estimates of area under the curve (AUC) based on validations of the fitted gradient boosted multiclass classifier (including age, sex and all eye movement features) on Test-1 (N=133) and Test-2[ǂ] (N=244) datasets

The corresponding model outcomes excluding age are presented in Table 2.

	**Reference**				**AUC[§]**			
**Prediction**	**CON**	**SCZ**	**BPAD**	**MDD**	**CON**	**SCZ**	**BPAD**	**MDD**
**Validation on Test-1 (N=133)**								
CON	**18**	3	4	5	**0.87**			
SCZ	7	**18**	5	3	0.86	**0.87**		
BPAD	5	3	**18**	7	0.85	0.82	**0.77**	
MDD	5	6	8	**18**	0.83	0.85	0.68	**0.76**
**Validation on Test-2 (N=244)**								
CON	**117**	22			**0.69**			
SCZ	16	**36**			0.80	**0.90**		
BPAD	29	2						
MDD	22	0						

[ǂ]The validation on Test-2 dataset does not have any representation of BPAD and MDD patients.
[§]AUC table represents the overall groupwise AUC by one-versus-all (OVA) (diagonal element) and pairwise AUCs by one-versus-one (OVO) methods (off-diagonal elements).
CON: Healthy Control; SCZ: Schizophrenia; BPAD: Bipolar Affective Disorder; MDD: Major Depression Disorder

Supplementary Table 7. Performance metrics based on the validation of the fitted gradient boosted multiclass classifier (including age, sex and all eye movement features) on Test-1 (N=133) and Test-2[ǂ] (N=244) datasets.

The corresponding model outcomes excluding age are presented in Table 3.

Statistics	CON	SCZ	BPAD	MDD
**Validation on Test-1 (N=133)**				
Sensitivity	0.51	0.60	0.51	0.55
Specificity	0.88	0.85	0.85	0.81
Positive Predictive Value (PPV)	0.60	0.55	0.55	0.49
Negative Predictive Value (NPV)	0.84	0.88	0.83	0.84
Precision	0.60	0.55	0.55	0.49
F1 Score	0.55	0.57	0.53	0.51
Accuracy	0.78	0.80	0.76	0.74
Balanced Accuracy	0.70	0.73	0.68	0.68
**Validation on Test-2 (N=244)**				
Sensitivity	0.64	0.60		
Specificity	0.63	0.91		
Positive Predictive Value (PPV)	0.84	0.69		
Negative Predictive Value (NPV)	0.36	0.88		
Precision	0.84	0.69		
F1 Score	0.72	0.64		
Accuracy	0.64	0.84		
Balanced Accuracy	0.63	0.76		

[ǂ]The validation on Test-2 dataset does not have any representation of BPAD and MDD patients.
CON: Healthy Control; SCZ: Schizophrenia; BPAD: Bipolar Affective Disorder; MDD: Major Depression Disorder

Supplementary Table 8. Detailed performance metrics along with 95% confidence interval (lower, upper)† based on validations of the fitted gradient boosted multiclass classifier (including age, sex and all eye movement variables) on Test-1 (N=133) and Test-2‡ (N=244) datasets

The corresponding model outcomes excluding age are presented in Supplementary Table 4.

Statistics	CON	SCZ	BPAD	MDD
**Validation on Test-1 (N=133)**				
AUC (OVA)§	0.87 (0.76, 0.92)	0.87 (0.75, 0.91)	0.77 (0.63, 0.83)	0.76 (0.63, 0.85)
AUC (OVO)§ with CON		0.86 (0.72, 0.91)	0.86 (0.75, 0.91)	0.83 (0.71, 0.89)
AUC (OVO)§ with SCZ			0.82 (0.67, 0.87)	0.85 (0.73, 0.91)
AUC (OVO)§ with BPAD				0.68 (0.52, 0.77)
Overall AUC§§	0.82 (0.73, 0.84)			
Sensitivity	0.51 (0.31, 0.71)	0.60 (0.33, 0.77)	0.51 (0.26, 0.66)	0.55 (0.30, 0.73)
Specificity	0.88 (0.80, 0.95)	0.85 (0.77, 0.93)	0.85 (0.71, 0.92)	0.81 (0.67, 0.89)
Positive Predictive Value (PPV)	0.60 (0.45, 0.78)	0.55 (0.38, 0.71)	0.55 (0.32, 0.66)	0.49 (0.30, 0.61)
Negative Predictive Value (NPV)	0.84 (0.79, 0.90)	0.88 (0.82, 0.93)	0.83 (0.75, 0.87)	0.84 (0.77, 0.90)
F1 Score	0.55 (0.39, 0.71)	0.57 (0.37, 0.70)	0.53 (0.29, 0.62)	0.51 (0.32, 0.63)
Balanced Accuracy	0.70 (0.60, 0.80)	0.73 (0.59, 0.82)	0.68 (0.54, 0.74)	0.68 (0.54, 0.76)

Statistics	CON	SCZ	BPAD	MDD
**Validation on Test-2 (N=244)**				
AUC (OVA)	0.69 (0.54, 0.77)	0.90 (0.80, 0.94)		

AUC (OVO) with CON		0.80 (0.68, 0.86)
Sensitivity	0.64 (0.54, 0.71)	0.60 (0.37, 0.75)
Specificity	0.63 (0.43, 0.80)	0.91 (0.87, 0.96)
Positive Predictive Value (PPV)	0.84 (0.77, 0.91)	0.69 (0.55, 0.84)
Negative Predictive Value (NPV)	0.36 (0.26, 0.43)	0.88 (0.82, 0.92)
F1 Score	0.72 (0.64, 0.78)	0.64 (0.45, 0.76)
Balanced Accuracy	0.63 (0.52, 0.72)	0.76 (0.64, 0.83)

[†]The bootstrap sampling with 1000 replicates from the relevant datasets was used to calculate the 95% confidence interval of different performance metrics.

[‡]The validation on Test-2 dataset does not have any representation of BPAD and MDD patients.

[§]AUC (area under the curve) represents the overall groupwise AUC by one-versus-all (OVA) and pairwise AUCs by one-versus-one (OVO) methods.

[§§]The overall AUC is the generalised overall AUC as proposed by Hand and Till (2001).

CON: Healthy Control; SCZ: Schizophrenia; BPAD: Bipolar Affective Disorder; MDD: Major Depression Disorder

Supplementary Table 9. Number of subjects for different levels of nicotine, caffeine and psychotropic medication and summary statistics (median, IQR) of HADS anxiety and HADS depression of different groups

Confounder	Levels	CON (N=177)	SCZ (N=150)	BPAD (N=176)	MDD (N=169)
Nicotine	0	141	63	111	107
	1-20	16	38	52	28
	20+	0	22	5	4
	NA	20	27	8	30
Caffeine	0	28	12	23	15
	1-5	109	71	111	79
	5-10	20	29	23	40
	10+	0	12	11	6
	NA	20	26	8	29
Medication	0	177	3	46	40
	1-300	0	37	46	10
	301-800	0	32	7	2
	800+	0	25	3	0
	NA	0	53	74	117
HADS Anxiety	Median (Q1, Q3)	4.0 (2.0, 6.0)	8.0 (6.0, 12.0)	8.0 (5.0, 12.0)	11.0 (7.0, 14.0)
HADS Depression	Median (Q1, Q3)	1.0 (0..0, 3.0)	7.0 (4.0, 9.2)	5.0 (2.0, 10.0)	8.0 (4.0, 11.2)

CON: Healthy Control; SCZ: Schizophrenia; BPAD: Bipolar Affective Disorder; MDD: Major Depression Disorder.

Medication Chlorpromazine (CPZ) equivalents (mg/day).

Supplementary Table 10. Principal component regression models of the first 20 PC scores and different confounding variables (N=672)

PC	Eigen	PC var	Cum PC var	Nicotine		Caffeine		Medication		HADS Anxiety		HADS Depression		Age	
				%	p-val	%	p-val	%	p-val	%	p-val	%	p-val	%	p-val
1	17.51	17.87	17.87	0.74	0.115	0.10	0.905	7.84	<0.001	0.10	0.461	0.49	0.106	9.57	<0.001
2	13.13	13.40	31.27	1.06	0.044	0.48	0.416	0.84	0.310	0.16	0.348	0.03	0.705	0.24	0.207
3	8.53	8.70	39.97	0.78	0.101	0.34	0.572	0.34	0.694	0.24	0.261	0.03	0.712	0.06	0.531
4	6.99	7.13	47.10	0.58	0.182	1.06	0.102	0.29	0.747	0.01	0.824	0.02	0.754	6.11	<0.001
5	4.74	4.83	51.94	0.51	0.225	0.50	0.406	4.65	<0.001	1.43	0.006	0.79	0.039	0.00	0.879
6	4.10	4.19	56.12	0.60	0.175	1.27	0.058	0.91	0.273	0.99	0.021	1.24	0.010	0.33	0.139
7	3.79	3.87	59.99	1.62	0.009	2.14	0.006	12.49	<0.001	0.04	0.635	0.06	0.562	1.66	0.001
8	2.18	2.23	62.22	0.01	0.959	0.79	0.198	3.82	0.001	0.58	0.078	1.64	0.003	0.00	0.987
9	2.08	2.12	64.34	0.65	0.150	1.61	0.023	0.49	0.555	0.04	0.646	0.05	0.622	0.01	0.754
10	1.96	2.00	66.34	0.25	0.475	0.17	0.796	1.49	0.095	2.08	0.001	1.66	0.003	0.91	0.014
11	1.65	1.69	68.03	0.16	0.630	0.19	0.781	4.95	<0.001	0.00	0.901	0.00	0.937	0.12	0.380
12	1.57	1.61	69.63	0.91	0.070	1.31	0.052	0.22	0.820	0.00	0.995	0.03	0.709	0.09	0.428
13	1.37	1.40	71.03	0.85	0.083	0.35	0.560	0.99	0.238	0.01	0.786	0.56	0.082	0.38	0.109
14	1.34	1.37	72.39	0.23	0.508	0.48	0.425	1.77	0.055	0.03	0.689	0.00	0.965	2.23	<0.001

15	1.30	1.33	73.72	0.27	0.459	0.56	0.352	1.05	0.213	0.27	0.231	0.03	0.711	0.01	0.832
16	1.23	1.25	74.97	0.20	0.560	2.89	0.001	1.06	0.210	0.00	0.923	0.03	0.681	0.01	0.761
17	1.09	1.11	76.08	0.03	0.912	0.53	0.373	0.18	0.853	0.00	0.894	0.37	0.158	0.09	0.427
18	1.04	1.07	77.15	0.21	0.543	0.62	0.306	0.77	0.349	0.16	0.356	0.25	0.245	0.00	0.892
19	1.00	1.02	78.17	2.82	0.000	0.87	0.164	1.33	0.129	0.10	0.467	0.12	0.433	0.71	0.029
20	0.95	0.97	79.15	0.23	0.509	0.33	0.584	2.15	0.027	0.03	0.691	0.35	0.174	0.06	0.513

PC: Principal Component (PC)

Eigen: The estimate of variance of the specified PC

PC var: The percentage of total variance explained by the specified PC

Cum PC var: The cumulative percentage of total variance explained by preceding PCs and the current PC

Confounding variables are nicotine, caffeine, psychotropic medication, anxiety and depression sub-scores from HADS questionnaire

%: The percentage of variance of the PC scores explained by the specific confounding variable

p-val: The p-value obtained from the fitted single variable principal component regression model

Supplementary Table 11. Observed and predicted class and prediction probabilities of different classes of individuals in the test dataset (N=133) based on the fitted gradient boosted multiclass classifier (including sex and all eye movement features)

Patient ID	Group	Predicted Class	Correct Prediction	Prob CON	Prob SCZ	Prob BPAD	Prob MDD
ID001	SCZ	SCZ	Yes	0.0015	0.8825	0.0483	0.0677
ID002	CON	SCZ	No	0.2155	0.3994	0.1225	0.2626
ID003	SCZ	MDD	No	0.2373	0.1763	0.0565	0.5298
ID004	CON	MDD	No	0.2380	0.0239	0.3250	0.4130
ID005	CON	CON	Yes	0.5707	0.0643	0.1913	0.1737
ID006	CON	MDD	No	0.2935	0.0138	0.0317	0.6610
ID007	CON	MDD	No	0.1252	0.0949	0.1191	0.6608
ID008	CON	BPAD	No	0.3632	0.0436	0.3824	0.2108
ID009	CON	CON	Yes	0.4962	0.0144	0.0168	0.4726
ID010	CON	CON	Yes	0.4874	0.0526	0.3470	0.1129
ID011	SCZ	CON	No	0.3023	0.2662	0.1475	0.2840
ID012	CON	MDD	No	0.1096	0.2493	0.0708	0.5703
ID013	SCZ	SCZ	Yes	0.0253	0.6269	0.1081	0.2397
ID014	SCZ	SCZ	Yes	0.0731	0.8431	0.0269	0.0570
ID015	SCZ	MDD	No	0.0765	0.3748	0.0304	0.5182
ID016	SCZ	MDD	No	0.0552	0.0304	0.1392	0.7751
ID017	SCZ	SCZ	Yes	0.0054	0.7912	0.1057	0.0977
ID018	SCZ	SCZ	Yes	0.0129	0.5293	0.2975	0.1603

ID019	SCZ	SCZ	Yes	0.0108	0.7963	0.1035	0.0894
ID020	SCZ	SCZ	Yes	0.0054	0.5072	0.2101	0.2772
ID021	CON	CON	Yes	0.9738	0.0041	0.0159	0.0061
ID022	CON	CON	Yes	0.9436	0.0019	0.0212	0.0333
ID023	CON	CON	Yes	0.4794	0.4091	0.0374	0.0740
ID024	CON	CON	Yes	0.6878	0.0248	0.0533	0.2341
ID025	CON	BPAD	No	0.0468	0.0164	0.7903	0.1465
ID026	CON	CON	Yes	0.5112	0.0154	0.4479	0.0256
ID027	SCZ	BPAD	No	0.1595	0.1321	0.4761	0.2323
ID028	SCZ	CON	No	0.8783	0.0728	0.0195	0.0294
ID029	CON	CON	Yes	0.7881	0.0068	0.1010	0.1041
ID030	CON	BPAD	No	0.0940	0.1751	0.5002	0.2307
ID031	SCZ	SCZ	Yes	0.0477	0.4295	0.2670	0.2558
ID032	SCZ	MDD	No	0.0248	0.3128	0.2436	0.4188
ID033	SCZ	SCZ	Yes	0.0870	0.5942	0.2105	0.1084
ID034	SCZ	BPAD	No	0.0034	0.3367	0.4037	0.2563
ID035	CON	SCZ	No	0.0533	0.5838	0.3507	0.0122
ID036	SCZ	CON	No	0.3459	0.1457	0.2955	0.2129
ID037	CON	CON	Yes	0.9610	0.0268	0.0075	0.0048
ID038	CON	CON	Yes	0.9814	0.0084	0.0024	0.0078
ID039	CON	CON	Yes	0.8453	0.1287	0.0206	0.0053

ID040	CON	CON	Yes	0.7469	0.1039	0.1160	0.0332
ID041	CON	CON	Yes	0.9971	0.0023	0.0004	0.0002
ID042	CON	CON	Yes	0.6207	0.3682	0.0093	0.0018
ID043	CON	CON	Yes	0.9489	0.0425	0.0061	0.0025
ID044	CON	SCZ	No	0.0284	0.8436	0.1093	0.0187
ID045	CON	SCZ	No	0.4570	0.4677	0.0592	0.0161
ID046	CON	BPAD	No	0.2650	0.0583	0.4748	0.2020
ID047	CON	SCZ	No	0.0065	0.6590	0.3016	0.0328
ID048	CON	SCZ	No	0.1559	0.5588	0.0316	0.2537
ID049	CON	CON	Yes	0.4861	0.0236	0.0285	0.4617
ID050	CON	CON	Yes	0.5714	0.0280	0.0430	0.3575
ID051	SCZ	SCZ	Yes	0.1075	0.7721	0.0852	0.0352
ID052	SCZ	SCZ	Yes	0.0244	0.5518	0.3565	0.0674
ID053	SCZ	MDD	No	0.1013	0.0818	0.2976	0.5194
ID054	SCZ	BPAD	No	0.0046	0.2193	0.5797	0.1963
ID055	SCZ	CON	No	0.8890	0.1052	0.0033	0.0024
ID056	SCZ	SCZ	Yes	0.0427	0.9537	0.0027	0.0008
ID057	SCZ	SCZ	Yes	0.0349	0.9549	0.0078	0.0023
ID058	SCZ	SCZ	Yes	0.4496	0.5448	0.0026	0.0030
ID059	SCZ	SCZ	Yes	0.0592	0.9196	0.0160	0.0053
ID060	SCZ	SCZ	Yes	0.0205	0.7152	0.1942	0.0702

ID061	SCZ	SCZ	Yes	0.0191	0.7130	0.2656	0.0023
ID062	BPAD	BPAD	Yes	0.1163	0.1923	0.4597	0.2318
ID063	BPAD	SCZ	No	0.0582	0.7788	0.0896	0.0734
ID064	BPAD	BPAD	Yes	0.0259	0.0362	0.5142	0.4237
ID065	BPAD	BPAD	Yes	0.2861	0.0809	0.6208	0.0122
ID066	BPAD	BPAD	Yes	0.0010	0.1952	0.6820	0.1218
ID067	BPAD	MDD	No	0.0165	0.3122	0.2303	0.4410
ID068	BPAD	BPAD	Yes	0.0194	0.0911	0.4915	0.3980
ID069	BPAD	MDD	No	0.0099	0.0262	0.2076	0.7563
ID070	BPAD	BPAD	Yes	0.0678	0.0061	0.8600	0.0661
ID071	BPAD	BPAD	Yes	0.0045	0.1069	0.4916	0.3969
ID072	BPAD	MDD	No	0.0037	0.0128	0.4071	0.5765
ID073	BPAD	CON	No	0.5975	0.0194	0.1510	0.2321
ID074	BPAD	CON	No	0.4494	0.0101	0.2383	0.3022
ID075	MDD	BPAD	No	0.0182	0.0191	0.4950	0.4677
ID076	MDD	BPAD	No	0.0147	0.3811	0.4060	0.1981
ID077	MDD	SCZ	No	0.0103	0.7222	0.2335	0.0340
ID078	MDD	MDD	Yes	0.0093	0.1373	0.2566	0.5968
ID079	MDD	BPAD	No	0.0016	0.0287	0.9258	0.0439
ID080	MDD	MDD	Yes	0.0093	0.0077	0.2582	0.7247
ID081	MDD	BPAD	No	0.0029	0.0026	0.8414	0.1532

ID082	MDD	BPAD	No	0.0126	0.0116	0.6634	0.3123
ID083	MDD	MDD	Yes	0.0054	0.2753	0.2115	0.5078
ID084	MDD	SCZ	No	0.0172	0.6097	0.2146	0.1585
ID085	MDD	CON	No	0.5791	0.0247	0.2961	0.1002
ID086	MDD	MDD	Yes	0.2601	0.1086	0.2497	0.3816
ID087	MDD	CON	No	0.5221	0.0117	0.3788	0.0874
ID088	MDD	MDD	Yes	0.1256	0.0511	0.1203	0.7030
ID089	MDD	CON	No	0.3900	0.1776	0.1488	0.2837
ID090	MDD	MDD	Yes	0.1267	0.0050	0.3794	0.4889
ID091	MDD	MDD	Yes	0.2456	0.0088	0.2313	0.5142
ID092	MDD	MDD	Yes	0.0473	0.0041	0.0969	0.8516
ID093	MDD	MDD	Yes	0.1338	0.0590	0.2416	0.5657
ID094	MDD	BPAD	No	0.0121	0.0139	0.5842	0.3898
ID095	MDD	MDD	Yes	0.0047	0.0126	0.1708	0.8120
ID096	MDD	MDD	Yes	0.0328	0.0021	0.0633	0.9018
ID097	MDD	MDD	Yes	0.0102	0.0375	0.4067	0.5456
ID098	MDD	MDD	Yes	0.0388	0.0039	0.0431	0.9142
ID099	MDD	MDD	Yes	0.0151	0.0110	0.0896	0.8844
ID100	CON	SCZ	No	0.1608	0.6392	0.0788	0.1213
ID101	CON	CON	Yes	0.9648	0.0073	0.0159	0.0120
ID102	CON	CON	Yes	0.5726	0.0812	0.0441	0.3021

ID103	BPAD	MDD	No	0.0219	0.1791	0.0859	0.7131
ID104	SCZ	SCZ	Yes	0.0218	0.8839	0.0556	0.0387
ID105	BPAD	MDD	No	0.0393	0.0694	0.1408	0.7506
ID106	MDD	CON	No	0.6021	0.0297	0.1670	0.2012
ID107	MDD	MDD	Yes	0.0545	0.0221	0.0351	0.8882
ID108	MDD	MDD	Yes	0.0323	0.0029	0.0189	0.9459
ID109	BPAD	BPAD	Yes	0.0907	0.0096	0.5095	0.3902
ID110	BPAD	BPAD	Yes	0.0729	0.0116	0.6592	0.2563
ID111	BPAD	MDD	No	0.0211	0.0920	0.3147	0.5722
ID112	MDD	CON	No	0.4480	0.1164	0.0475	0.3880
ID113	BPAD	CON	No	0.4512	0.0457	0.3150	0.1881
ID114	MDD	SCZ	No	0.0740	0.7122	0.1086	0.1052
ID115	BPAD	BPAD	Yes	0.0125	0.1303	0.7483	0.1089
ID116	BPAD	BPAD	Yes	0.0523	0.3067	0.4830	0.1580
ID117	MDD	CON	No	0.5711	0.0080	0.0773	0.3436
ID118	BPAD	SCZ	No	0.0071	0.6971	0.0709	0.2249
ID119	BPAD	BPAD	Yes	0.0091	0.1036	0.8201	0.0672
ID120	MDD	MDD	Yes	0.1483	0.3522	0.1029	0.3966
ID121	BPAD	SCZ	No	0.0130	0.6837	0.1196	0.1837
ID122	BPAD	MDD	No	0.1378	0.2407	0.2651	0.3564
ID123	BPAD	SCZ	No	0.0050	0.7769	0.1582	0.0599

ID124	BPAD	CON	No	0.7973	0.0245	0.1543	0.0240
ID125	BPAD	BPAD	Yes	0.0196	0.1290	0.6598	0.1916
ID126	BPAD	BPAD	Yes	0.0483	0.0339	0.8356	0.0822
ID127	BPAD	BPAD	Yes	0.1367	0.0099	0.5999	0.2534
ID128	BPAD	BPAD	Yes	0.0325	0.0035	0.9201	0.0440
ID129	BPAD	MDD	No	0.0450	0.0327	0.3335	0.5887
ID130	BPAD	BPAD	Yes	0.3780	0.0758	0.4644	0.0818
ID131	BPAD	SCZ	No	0.0242	0.4381	0.4301	0.1076
ID132	BPAD	MDD	No	0.0751	0.2425	0.1270	0.5554
ID133	MDD	MDD	Yes	0.0650	0.0354	0.0644	0.8352

CON: Healthy Control; SCZ: Schizophrenia; BPAD: Bipolar Affective Disorder; MDD: Major Depression Disorder

The column "Correct Prediction" indicates if the model predicted class is identical (Yes) or different (No) to the observed class (Group)

Columns with Prob CON, SCZ, BPAD and MDD indicate the probabilities of the corresponding class as produced by the fitted gradient boosted model (including all eye movement features and sex) on the Test-1 dataset (N=133).

References

(1) Maros ME, Capper D, Jones DTW et al Machine learning workflows to estimate class probabilities for precision cancer diagnostics on DNA methylation microarray data. Nature Protoc. 2020; **15:**479-512.

(2) Platt, J. Probabilistic outputs for support vector machines and comparisons to regularized likelihood methods. Adv. Large Margin Classifiers 1999; **10:** 61–74.

(3) Firth, D. Bias reduction of maximum likelihood estimates. Biometrika 1993; **80:** 27–38.

(4) brglm: Bias Reduction in Binary-Response Generalized Linear Models. R package version 0.7.1, 2020; https://cran.r-project.org/package=brglm.

(5) Friedman J, Hastie T, Tibshirani R. "Regularization Paths for Generalized Linear Models via Coordinate Descent." Journal of Statistical Software 2010; **33:**1–22.

(6) Hand DJ and Till RJ. A simple generalisation of the area under the ROC curve for multiple classification problems. Mach. Learning 2001; **45:**171-186

(7) Chen, T and Guestrin, C. XGBoost: A Scalable Tree Boosting System. In 22nd SIGKDD Conference on Knowledge Discovery and Data Mining, 2016.

(8) xgboost: Extreme Gradient Boosting. R package version 1.3.2.1, 2020; https://cran.r-project.org/package=xgboost.

(9) R Core Team. R: A language and environment for statistical computing. R Foundation for Statistical Computing, 2020; Vienna, Austria.
